# Intercalation Favors DNA Covalent Photobinding in Photoresponsive Dual PDT/PCT Bimetallic Assemblies

**DOI:** 10.1021/acs.jcim.6c00922

**Published:** 2026-05-29

**Authors:** Abdelazim M. A. Abdelgawwad, Daniel Roca-Sanjuán, Marta E. Alberto, Antonio Francés-Monerris

**Affiliations:** † Institut de Ciència Molecular, 16781 Universitat de València, P.O. Box 22085, València 46071, Spain; ‡ Dipartimento di Chimica e Tecnologie Chimiche, Università della Calabria, Arcavacata di Rende I-87036, Italy

## Abstract

The local treatment of solid tumors through photoactivated therapies demands the development of alternative strategies independent of oxygen levels, which are often very low in cancerous tissues. In this regard, the combination of an efficient reactive oxygen species (ROS) photogenerator with a drug that covalently targets DNA represents a valuable approach due to the *in situ* combination of type I/II photodynamic reactions with the covalent blockage of the DNA biological function. In this context, the theoretical framework of the chemical events that cause the observed phototoxicity is far from being fully understood, especially the dynamic factors, timescales, and environmental effects. This work sheds light on the molecular basis of these events by studying the DNA photoreactivity of a Ru­(II)/Os­(II) and a Pt­(II) bimetallic assembly via microsecond molecular dynamics and multiscale biased quantum mechanics/molecular mechanics (QM/MM) MD simulations. Analysis of the DNA interaction modes reveals persistent major/minor groove interactions of the photosensitizer and a thermodynamically favored DNA intercalation. On the other hand, the free energy landscapes reveal kinetically fast (energy barriers *ca.* 6 kcal·mol^–1^) ligand exchange reactions between the N7 position of guanine and the platinum center in the triplet excited state, clearly highlighting the role of light in accelerating the chemical process. Additional analyses suggest that DNA intercalation, often associated with high cellular toxicity, could instead be seen as an opportunity to increase phototoxicity indexes by reducing the DNA conformational space available for photoreactions and improving absorption properties.

## Graphical abstract

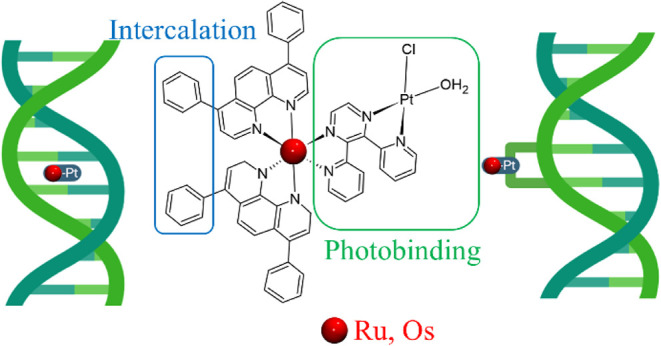

## Introduction

In the continuous quest for novel drugs capable of enhancing the therapeutic efficacy of current anticancer approaches, the integration into the same chemical structure of two or more therapeutic agents to yield an enhanced therapeutic effectiveness represents an emerging area of interest.^1–4^ The main advantage of these multi-targeted agents relies on the possibility to combine several pharmacological responses arising from the retained ability of each component to interact with its specific target site(s).

Several examples of two-component assemblies have been proposed and explored in the last decade with the primary goal to reach a therapeutic efficacy exceeding that of the individual precursors by exploiting the synergy between the two components.^5^ The combination of a photodynamically active photosensitizer (PS) with a DNA-targeting Pt­(II)-moiety ensures the integration of photodynamic therapy (PDT) with chemotherapy for a synergistic anticancer effect.^6–8^ In addition to leveraging the two primary treatment modalities, these kinds of multi-component agents are conceived to overcome some of the most severe limitations of PDT. One of the PDT major drawbacks arises from its high dependence on oxygen, whose levels are often poor in solid tumors due to the development of hypoxia conditions. Moreover, the non-homogeneous distribution of the PS inside the target tissue frequently prevents complete eradication.^9^ To mitigate these issues, the approach harnesses the ability of the Pt­(II)-bioactive site to target DNA to ensure a better distribution of the PS within the tumor, acting, at the same time, as a ROS generator. More importantly, the intrinsic oxygen-independent cytostatic activity exerted by the Pt­(II) center helps to ensure the efficacy of the assembly even at a low oxygen tension. Some noteworthy examples of two-component systems following this strategy are available in literature, in which both organic and metal-based chromophores were used in combination with DNA-oriented agents.^10–12^

Ru­(II)-polypyridil complexes are among the most promising PSs due to their high efficiency in the field of PDT^13–19^ and for which the interest has been further boosted with the recent advancement of TLD-1433 (Ruvidar®) in Phase II clinical trials for the treatment of non-muscle invasive bladder cancer.^20,21^ Some Ru­(II)/Ru­(III) centers coupled with Pt­(II)/Pt­(IV) complexes existing in literature^10,22,23^ have proved to enhance the anticancer efficacy of platinum drugs and overcome their shortcomings. Among them, intriguing features have been described by Brewer and coworkers, initially for the monocomponent Ru- and Os- [(Ph_2_phen)_2_M­(dpp)]^2+^ (Ph_2_phen = 4,7-diphenyl-1,10-phenanthroline, also known as DIP; dpp = 2,3-bis­(2-pyridyl)­pyrazine; M= Ru/Os) polyazine complexes, which showed light-promoted cytotoxicity in rat malignant glioma F98 cells.^24,25^ The heterometallic **Ru-Pt** assembly based on the combination of [(Ph_2_phen)_2_Ru­(dpp)]^2+^ with a PtCl_2_ subunit via the rigid dpp bridging ligand was reported afterward (Scheme 1). The enhanced cytotoxicity found for the **Ru-Pt** mixed complex was attributed to the dual toxicity pathways exerted by the assembly.^26,27^ Thereby, the DNA binding ability of the platinated subunit adds to the oxidative stress induced by the Ru polypyridil chromophore due to PDT mechanisms.^26,27^ The same authors demonstrated that, in F98 malignant glioma cells, visible light irradiation led to enhanced cellular uptake and antiproliferative activity compared with standalone *cis*-Pt. In addition, the selective DNA cleavage, compared with other proteins such as bovine serum albumin (BSA), indicates a potential for reduced off-target binding in vivo and diminished side effects compared with existing platinum-based therapeutics.^27^

**Scheme 1:**
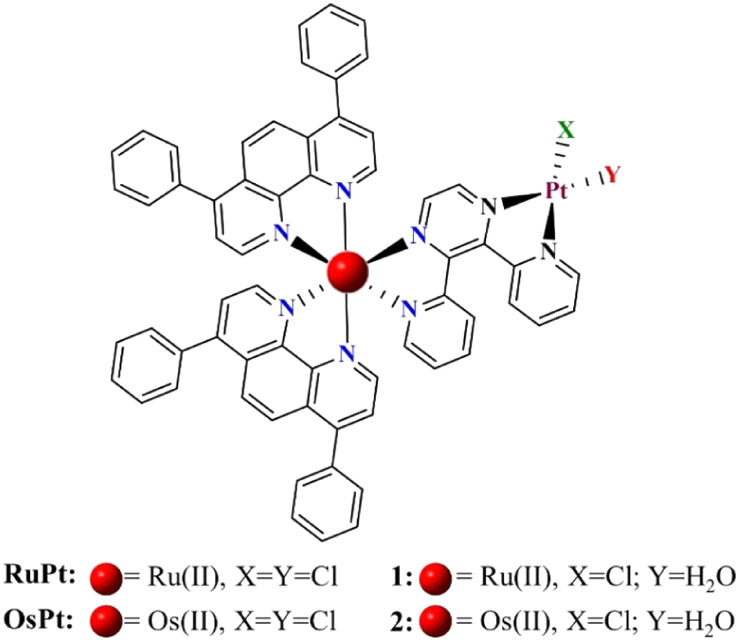
**Ru-Pt**, **Os-Pt** Complexes and Their Mono-Hydrated Derivatives **1** and **2,** Respectively

Theoretical studies carried out on both the **Ru-Pt** and **Os-Pt** complexes (Scheme 1) have clearly highlighted a synergistic effect between the two metal centers that underpin the greater efficacy of these conjugates^28,29^ compared with the monometallic precursors.^25^ In particular, the incorporation of platinum into the **Ru-Pt** assembly inducing a red shift of the key Ru→dpp metal-to-ligand charge transfer (MLCT) absorption band, extending it into a more tissue-penetrant spectral window, was rationalized from a quantum-chemistry perspective.^29^ Moreover, the presence of Pt markedly enhances spin-orbit coupling (SOC), thereby promoting efficient intersystem crossing to the triplet excited state. These photophysical modifications favor a Type II photodynamic mechanism, in which energy transfer occurs from the Ru-based triplet excited state (^3^MLCT) to molecular oxygen (^3^O_2_), generating singlet oxygen (^1^O_2_). On the other hand, the presence of the Ru/Os scaffold was ascertained to enable a novel mode of Pt-moiety photoactivation through light-induced release of the chloride leaving group in the excited state.^28,29^ In combination with Pt­(II)-ligands, the use of metallic photosensitizers emerged as indispensable requirement to reach a significant dual activity, since the presence of two metals proved essential for maximizing the synergistic effects.^28,29^

The key role of a ^3^MLCT excited state in promoting the labilization of Pt-Cl bond and the consequent DNA binding has been clearly established from both experiments and theory.^26,28,29^ However, the detailed sequence of events by which these ^3^MLCT states deliver the photodamage to cellular macromolecules remains unknown. These paths are different to those of cisplatin and other classical Pt­(II)-drugs in the dark, for which the Pt-Cl bonds undergo aquation once the complex enters the cytosol, where the chloride concentration is relatively low and water molecules are abundant.^30–33^ This substitution reaction generates the hydrolyzed Pt species, which represents the active form capable of penetrating the nuclear membrane to bind DNA at nucleophilic sites, most notably the N7 position of guanine, in the ground state.^33^ These bimetallic assemblies thus represent a rare example of a Pt­(II)-photobinding agent whose mechanism demands a deeper inspection.

This work characterizes the DNA covalent photoreaction of the hydrolyzed triplet excited state of both RuPt and OsPt complexes **1** and **2**, respectively (Scheme 1), through a multiscale approach based on a combination of molecular dynamics (MD) and quantum mechanics/molecular mechanics (QM/MM) MD simulations. Note that only **1** has been synthesized in the literature,^26^ whereas the Ru→Os replacement in **2** may offer additional advantages; however, it remains a purely theoretical model, as discussed elsewhere.^28^ Our findings indicate that both the Ru,Os-Pt complexes integrate structural disruption via DNA photobinding (DNA intra-strand cross-link) and intercalation mechanisms with the oxidative stress promoted by the metallic light absorbers, which confirms the coexistence of different cytotoxic mechanisms and rationalizes the dual PDT/photochemotherapy (PCT) activity.

## Computational Details

Complexes **1** and **2** have been parameterized by means of the easyPARM tool^34,35^ employing quantum mechanical (QM) data obtained at the DFT/B3LYP in combination with the 6–31G* basis set for all atoms except metals, which were described with the quasi-relativistic Stuttgart-Dresden (SDD) pseudopotential.^36^ Atomic partial charges were derived using the restrained electrostatic potential (RESP) method as implemented in AmberTools,^37^ computed at the same level of theory. All quantum mechanical (QM) calculations were performed using the Gaussian 16 software package without any symmetry restraint.^38^

The initial B-DNA double helix structure with the sequence **A** (Figure 1) was generated using the NAB utility of the AmberTools software package.^37^ This sequence was chosen as a DNA double helix representative model based on previous literature,^39,40^ whereas **B** represents the A→C mutation at position 10 to facilitate the full formation of a DNA intrastrand cross-link (N7 positions of guanine are deemed as one of the most nucleophilic DNA hotspots^33,39^ and **A** does not possess adjacent guanine bases). Water molecules and Na^+^ counter ions were described with the TIP3P parameters, whereas the DNA was described using the parm99 force field^41^ with bsc1 corrections.^42^ Starting from random positioning of the bimetallic assemblies, three independent production dynamics were run under NPT conditions for a total simulation time of 1 μs per replica at 310.15 K. To study the intercalation of the DIP ligand between adjacent nucleobases, three additional 1-μs replicas were carried out starting by manually positioning the complex between two adjacent DNA base pairs. Additionally, 1-μs simulation was carried out using an alternative initial orientation of complex **1**. To further assess long-timescale stability, one replica was extended by an additional 1-μs, resulting in a total simulation time of 2-μs. Intercalation free energy for complex **1** was estimated using the molecular mechanics Poisson-Boltzmann surface area (MM-PBSA) approach,^43^ validated with the available experimental data for the reference complex [Fe­(phen)­(DIP)_2_]^2+^.^44^ All molecular dynamics (MD) simulations were performed using the Amber 22 software package.^45^ Trajectories were analyzed with the MDanalysis^46^ and visualized through VMD.^47^

**Figure 1:**
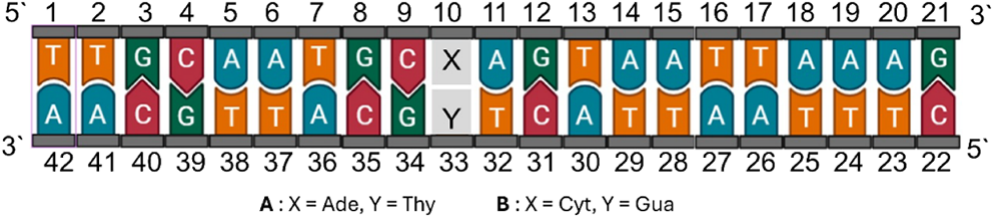
DNA sequences **A** and **B** and nucleotide numbering.

Representative snapshots capturing the major groove and intercalation interactions between the complex and DNA were extracted from the MD simulations for QM/MM reactivity, which was studied by means of the Amber 22/ORCA 6.1 interface^48^ and the electrostatic embedding.^45^ As validated in a previous work,^28^ the QM region of the mono-adduct (Figure 2) was treated using the M06 functional combined with the 6–31G basis set for all atoms except Os, Ru, and Pt, which were modeled using the SDD basis set. The ground state (S_0_) was treated with restricted DFT/M06, while the triplet state (T_1_) was treated using the unrestricted UDFT/M06 method. The QM partition (Figure 2) comprised the metal complex and the interacting nucleobase, with the QM/MM boundary defined across the nucleobase sugar covalent bond. Boundary bonds were capped by hydrogen atoms using the link atom approach as implemented in Amber, in which hydrogen link atoms are placed along the cut bond vector to saturate the QM valence. Bidirectional QM/MM relaxed scans were employed to generate initial umbrella sampling^49^ seeds. Afterward, 18–25 ps production QM/MM MD were run to sample the Pt-N7 reaction coordinates (Tables S1-S5 list the specific windows and simulation times, whereas Figures S1-S5 show the overlap between windows). To assess convergence, PMF was reconstructed excluding the last 5 ps of each umbrella sampling window. As shown in Figure S6, the PMF profile, particularly in the barrier region (2.0–4.0 Å), is well converged, indicating that the calculated free-energy barriers are robust with respect to the sampling length.

**Figure 2:**
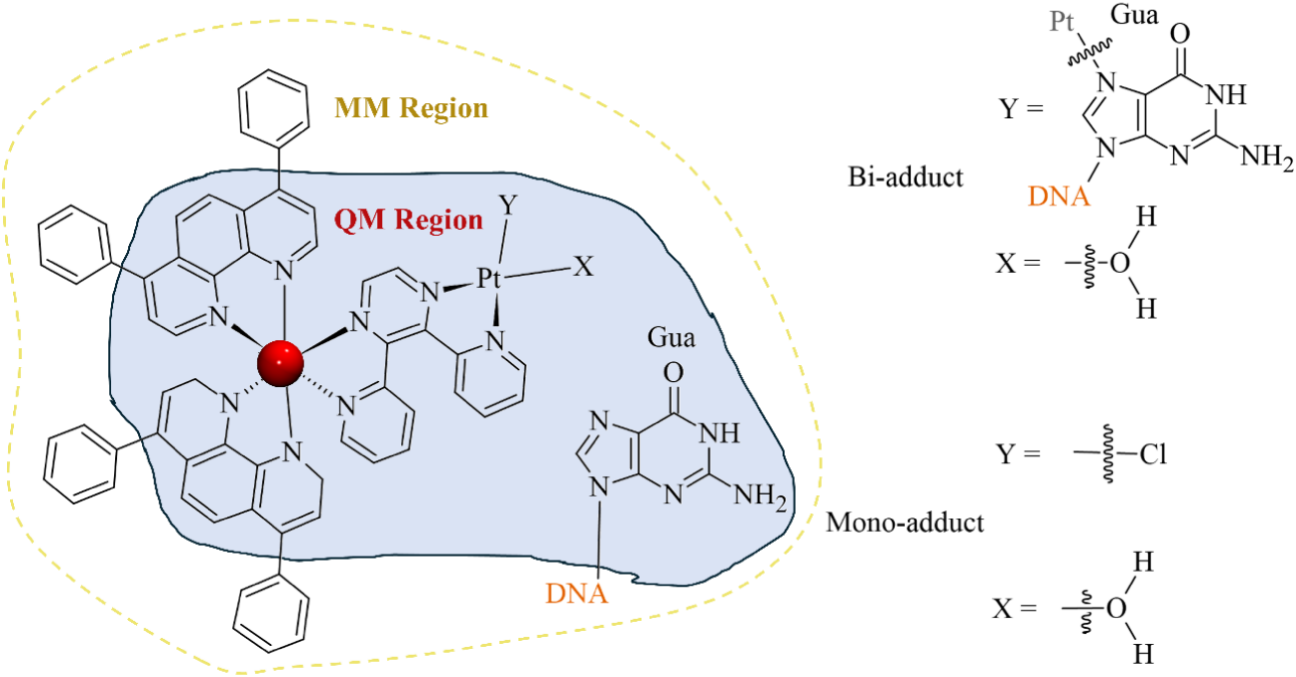
Schematic representation of the QM/MM partitioning employed in this study, with the QM region highlighted in blue. Gua denotes the guanine nucleobase. Two distinct QM regions were defined: the monoadduct QM region, used to model formation of the first Pt-N bond, and the bi-adduct QM region, employed to describe formation of the second Pt-N bond leading to the 1,2-intrastrand cross-link. The MM region comprises the remaining DNA framework, solvent molecules, and counterions.

To evaluate the effect of DNA intercalation on the electronic absorption spectrum of complex **1**, the UV-Vis spectrum of the DNA/**1** system and of **1** in pure water solution (hereafter, water/**1**) was computed by means of QM/MM methodology extracting 100 equally spaced snapshots from the MD simulations. The QM region of DNA/**1** included the metal complex and the four nucleobases surrounding the intercalation site, namely Adenine 10 and 11 and Thymine 32 and 33. For water/**1**, the QM region contained only complex **1**. The S_0_ state of each snapshot was equilibrated during 100 steps by means of QM/MM MD using the Amber/Terachem interface,^50–52^ whereas vertical excitation energies and oscillator strengths made use of the Amber/Gaussian 16 interface and the TD-M06/6–31+G­(d,p)/SDD/MM level of theory, a method widely validated in the literature.^28^ Cross sections (σ) were computed using the code MULTISPEC, developed in our group.^53^

The reader is referred to the Supporting Information for the full computational details.

## Results and Discussion

### Photoinduced Covalent Bond in the DNA Major Groove

Motivated by experimental evidence indicating that Ru-Pt assemblies can photobind DNA upon population of a ^3^MLCT state,^27^ previous theoretical studies have investigated the photoinduced labilization of the Pt-Cl ligand in the Ru,Os-Pt bimetallic complex.^28,29^ The QM/MM MD activation free energy associated with chloride dissociation and its replacement by a water molecule was estimated to be 3.4 kcal·mol^–1^,^28^ suggesting a kinetically fast and biologically accessible process, representing an alternative and rather unique mode of activation for such complexes prior to reaching their biological target. In *cis*-platin, the prototypical example, the hydrolysis of Pt-Cl bonds is favored in the intracellular media due to its low chloride concentration^33^ before the drug penetrates the nuclear membrane and reaches DNA.^54–56^ Consequently, this work considers that the Pt centers of the phototherapeutic agents are already partially hydrolyzed when interacting with DNA. Note that the lower intracellular Cl^–^ concentration relative to the higher extracellular one favors the classical ground-state activation mechanism even in the dark, in analogy with classical Pt­(II) drugs.

Regardless of the reaction kinetics, the chloride–water ligand exchange is expected to occur outside the nucleus and before the activated drug reaches nuclear DNA. The purpose of the current investigation is to extend the previously investigated hydrolysis process,^28,29^ which nevertheless forms the necessary foundation of the present work, by addressing the subsequent molecular events responsible for biological activity.

Visual inspection of the multiple replica simulations of complex **1** with DNA indicates that the bimetallic complex establishes spontaneous and stable non-covalent interactions with DNA. As shown in Figure 3, these interactions occur through both minor- and major-groove binding modes, with the hydrolyzed Pt fragment preferentially engaging nucleophilic sites located in the grooves, in particular, the N7 positions of guanine and adenine bases. Analysis of the distances between the Pt center and the N7 atoms of all guanine and adenine residues within the DNA (Figures S7-S9) quantify the interaction mechanism. It can be clearly seen that, in all the three replicas, the platinum atom remains close to the N7 positions (distances ∼6 Å) for several tens of nanoseconds. These persistent interactions are fully consistent with previous studies demonstrating that cisplatin targets N7 positions to form intrastrand cross-links.^33,56^

**Figure 3:**
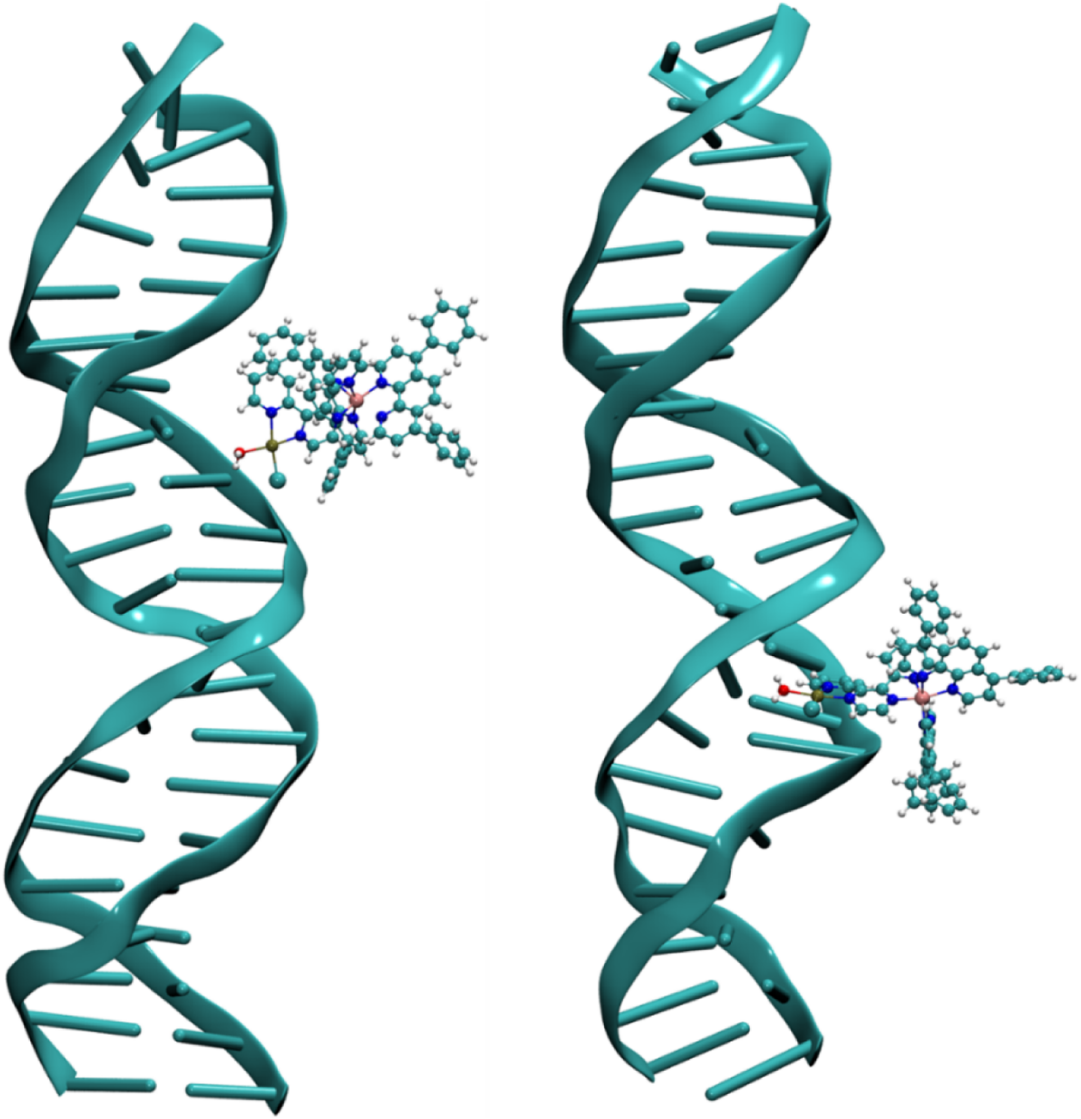
Representative MD snapshots depicting the major- and minor-groove interactions between complex **1** (balls and sticks) and DNA **A** (ribbons).

The mechanistic pathway underlying the photoreaction between the hydrolyzed Pt fragment of complexes **1** and **2** and DNA, ultimately leading to the formation of 1,2-intrastrand cross-links, is studied in the following. This mechanism can be seen as a ligand exchange between the water molecule coordinating the Pt center and DNA accelerated by the excited triplet state population. Notably, this mechanism differs from that of conventional Pt­(II) anticancer agents, for which DNA coordination typically proceeds via ground-state activation rather than photoinduced pathways.^30,33^

Figure 4A plots the free energy surface along the Pt-N7 reaction coordinate for both complexes obtained via biased QM/MM MD simulations (umbrella sampling), which represent the ligand exchange of one H_2_O molecule by DNA through the N7 position of guanine. The Pt center of complexes **1** and **2** (Scheme 1) becomes available to coordinate nucleophilic sites on DNA **A** (Figure 1), most prominently the N7 position of guanine (in this case, Gua 34), which is well known for its high electron density and strong affinity toward platinum-based electrophiles.^33,56^ At large Pt-N7 distances >4.5 Å, the potential of mean force (PMF) profile is relatively flat, with minor fluctuations attributable to steric constraints imposed by the environment and DNA groove. In the triplet state (T_1_), the Pt atom approach toward the N7 site is associated with a moderate free-energy barrier of approximately 6.2 kcal·mol^–1^ for complex **1** and a slightly higher value (∼6.7 kcal·mol^–1^) for complex **2**, indicating that the monoadduct formation is energetically accessible at biological temperatures. In contrast, the free energy barrier for the DNA coordination to **1** in the dark (ground state, S_0_) is twice that of T_1_ 12.25 kcal·mol^–1^, clearly demonstrating the acceleration caused by light.

**Figure 4:**
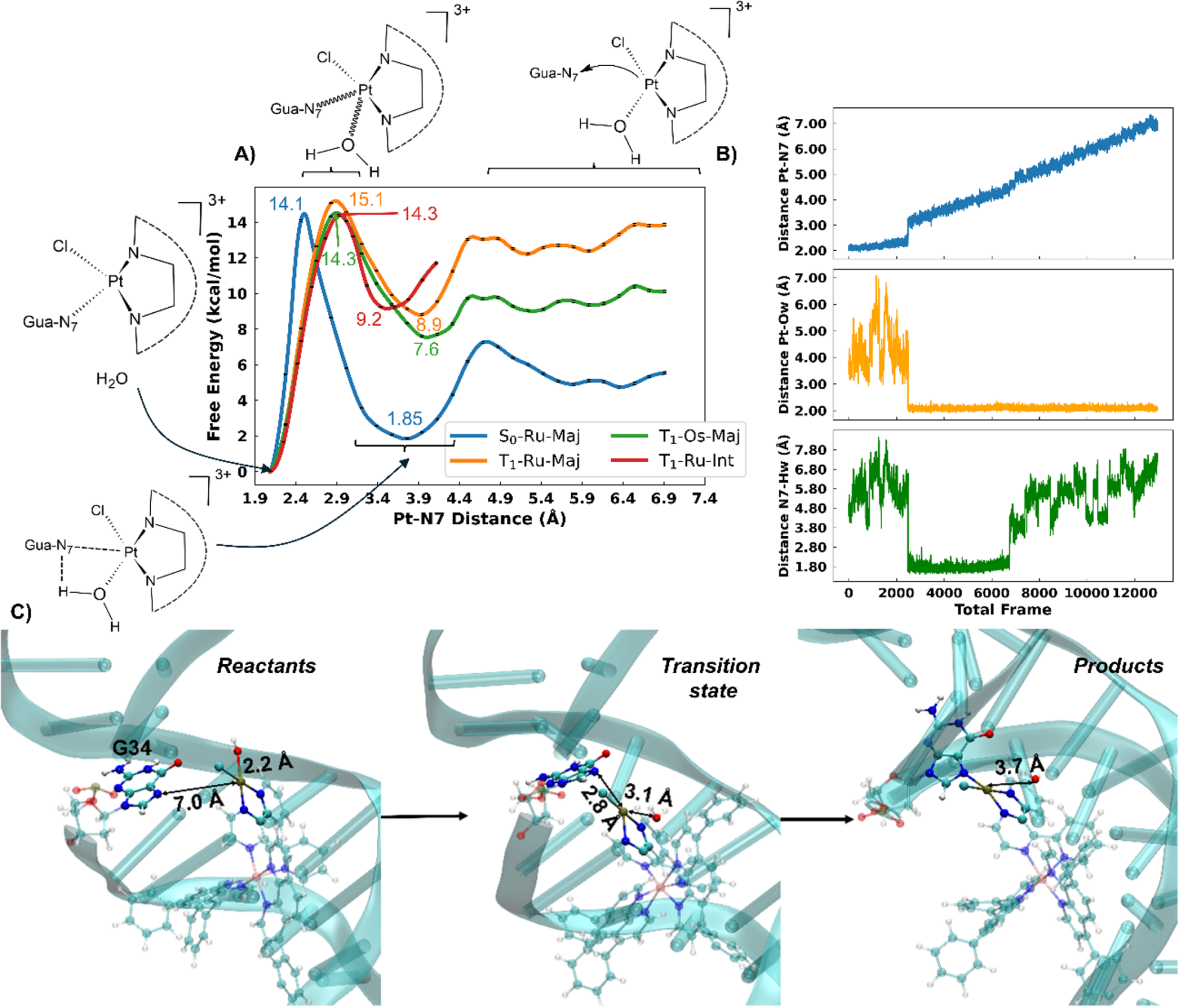
QM/MM reactivity of complexes **1** and **2** with DNA **A**. A) PMF profiles for Pt-N7­(Gua34) reaction coordinate in the first triplet state (T_1_) and ground state (S_0_), computed with the weighted histogram analysis method (WHAM).^57^ B) Pt-N7, Pt-Ow, and N7-Hw distances extracted from QM/MM MD simulation windows for complex **1** + **A** in the triplet state. Total frame refers to the sum of all frames for each window. C) Representative QM/MM snapshots extracted from QM/MM umbrella sampling windows. Gua-N7 refers to the N7 position of guanine.

The free-energy minimum between 3.9 and 4.5 Å region is attributed to the hydrogen bonding between the water hydrogen (Hw) and the N7 atom of guanine (frames ∼2500 to ∼7000 in Figure 4B, a balls and sticks model is shown in Figure S10). This H bond stabilizes pre-reactive configurations, facilitates the proper orientation of the Pt center relative to the nucleobase, and its necessary disruption to release the water molecule and coordinate DNA contributes to the free energy barrier. The transition region, characterized by a Pt-N7 distance of ∼2.8–2.9 Å in the triplet state and ∼2.35–2.45 Å in the ground state, is associated with partial weakening of the Pt-Ow dative bond, consistent with a ligand-exchange-like transition state. Finally, both complexes display a well-defined free-energy minimum at short Pt-N7 distances (∼2.1 Å), corresponding to the formation of a stable Pt-guanine coordination bond and defining the bound basin of the PMF. Selected snapshots shown in Figure 4C illustrate the key stages of this reaction.

For comparison, the activation barrier for the ground-state reactivity of the first substitution of *cis*-platin with DNA has been reported to be as high as 19.5 (B3LYP)^58^ and of 18.3 (liquid chromatography)^59^ kcal·mol^–1^. Similar results are obtained for related derivatives.^30^ Here, the S_0_ barrier is 12.25 kcal·mol^–1^. Although smaller, comparisons are reasonably in good agreement considering the differences in the molecular structure, the computational methodology employed with respect to previous theoretical works based on static profiles, and the incomplete sampling limited by QM/MM computational cost, which prevents the exploration of multiple initial reactants configurations.

The relatively low energy barriers (6.2 and 6.7 kcal·mol^–1^ for **1** and **2**, respectively) therefore account for the influence of light absorption and triplet state population in accelerating the kinetics of the DNA covalent binding. Under pseudo-first-order conditions, the rate constants can be estimated with the Eyring equation and the transition state theory, k=kBThe−ΔGRT , where *k_B_*, *h*, and *R* are the Boltzmann, Planck, and ideal gas constants, and Δ*G* is the free energy barrier. Reaction times can be estimated with *τ* = 1/*k*, giving values of 4, 8 ns, and 67 μs, respectively. These results clearly indicate that the photoexcited (triplet) pathway is several orders of magnitude faster than the ground-state reaction, and also faster than the non-covalent DNA/**1** groove dynamics (tens to hundreds of nanoseconds), thereby favoring the reactive process.

Photoexcitation markedly facilitates covalent DNA coordination of **1** in good agreement with experimental observations, where upon photoexcitation, population of the Ru→dpp ^3^MLCT state enhances electron density on the dpp ligand coordinated to Pt, thereby reducing the Lewis acidity of the platinum center. This electronic redistribution^26,28^ promotes chloride photolabilization, subsequent hydrolysis to the hydrolyzed species, and ultimately, rapid covalent attack on the DNA base, as shown in this work. The Ru→Os replacement does not substantially affect the kinetics of the photoprocess.

Figure 5A reports the free-energy profile associated with coordination of the Pt center of the monoadduct **1**/Gua34 to the adjacent guanine base Gua 33 (sequence **B**, Figure 1) to complete the 1,2-intra-strand DNA cross-link. The mechanistic features of this second coordination step are very similar to those observed for the monoadduct formation; however, the reaction occurs from a pre-organized state in which the platinum center is already pre-coordinated to DNA, resulting in a shorter initial Pt-N7­(Gua33) separation. The free-energy barrier for formation of the second Pt-N bond is reduced to 4.89 kcal·mol^–1^, lower than that associated with the initial monoadduct formation, leading to an estimated reaction time of 0.5 ns through the Eyring equation. This decrease might be attributed to the DNA/platinum center preorganization, driven by the previously formed Pt-N7 (Gua34) covalent bond (see Figure 5C), that decreases the energy penalty in the transition state. As expected, the Pt-N7 (Gua34) bond remains intact throughout this second concerted ligand exchange (Figure 5B), while the Pt-N7­(Gua33) reaction coordinate labilizes the Pt-Ow bond. Progression along the reaction coordinate leads to a transition region characterized by partial formation of the second Pt-N7 bond at approximately 2.89 Å, concomitant with elongation of the Pt-Ow bond. Completion of this step yields the 1,2-intrastrand cross-linked adduct, with both guanine bases coordinated to the platinum center and the water molecule fully displaced.

**Figure 5:**
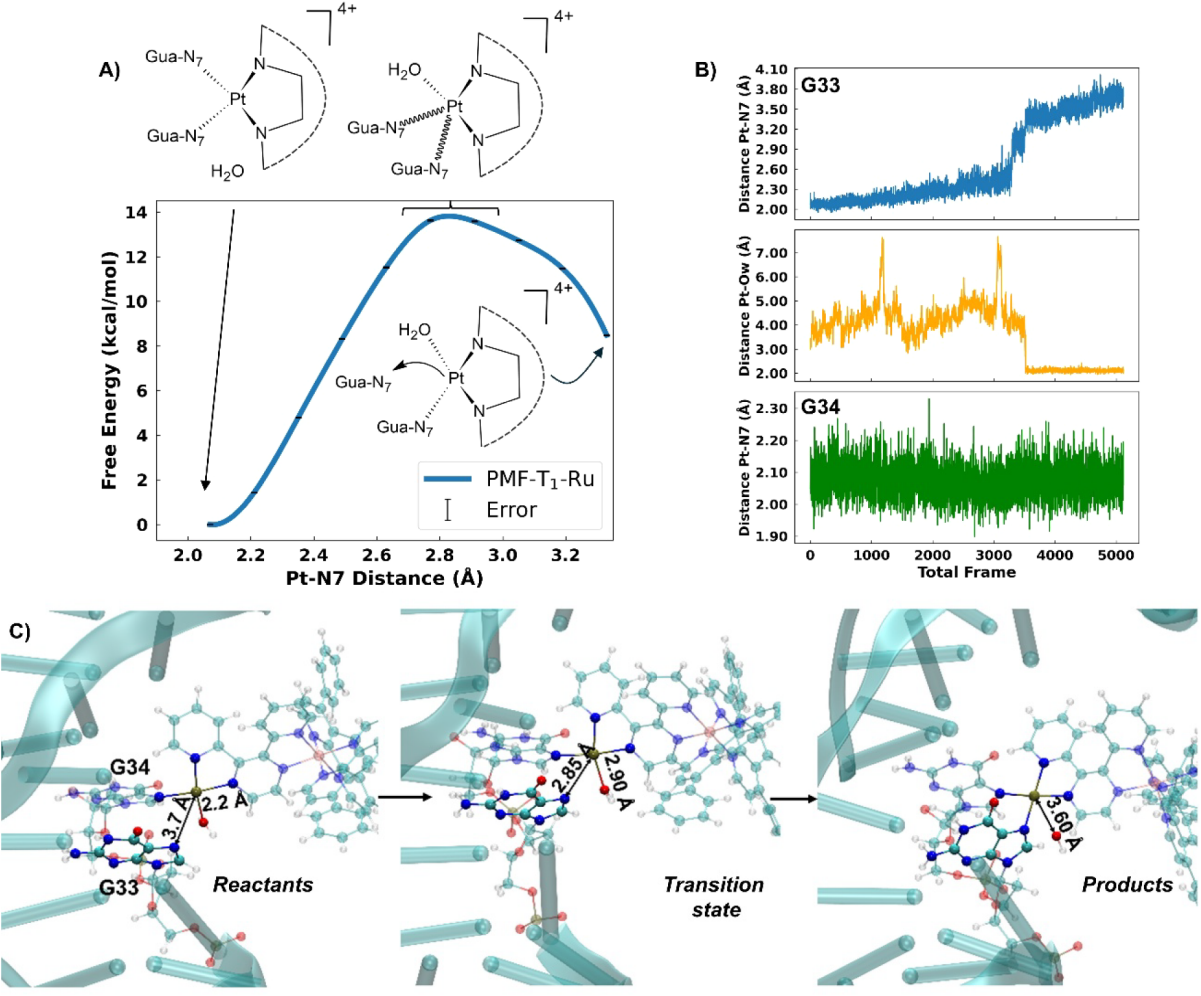
QM/MM reactivity between the monoadduct of complex **1** with DNA **B**. A) PMF profiles for Pt-N7­(Gua33) reaction coordinate in the first triplet state (T_1_), computed with the weighted histogram analysis method (WHAM).^57^ B) Pt-N7­(Gua33), Pt-Ow, and Pt-N7­(Gua34) distances extracted from QM/MM simulation windows for monoadduct **1** + **B**. Proposed concerted mechanism for formation of the second Pt-N7 bond leading to the 1,2-intrastrand cross-linked adduct. C) Representative QM/MM snapshots extracted from QM/MM umbrella sampling windows. Gua-N7 refers to the N7 position of guanine.

### DNA Intercalation: Impact on the Double-Strand Structure and Implications for DNA Covalent Photobinding

The planarity of the DIP ligands suggests that, besides the groove interaction spontaneous in our simulation timescales (a few μs), DNA intercalation could also play a very important role in the nucleic acid reactivity. As a matter of fact, there is evidence in the literature that points in this direction. Mudasir et al. studied the iron-based complex [Fe­(phen)­(DIP)_2_]^2+^, structurally related to **1**, reporting a DNA binding free energy (Δ*G*) of −24.7 kJ·mol^–1^ (−5.90 kcal·mol^–1^).^44^ The presence of DIP ligands was deemed as crucial to enhance DNA-binding affinity, an effect attributed to intercalation of the phenyl substituents of the DIP ligand between DNA base pairs. Similarly, Shahabadi et al. reported that the PtCl_2_(DIP) complex intercalates to calf thymus DNA throgh the DIP ligand.^60^ Moreover, several studies have shown that Ru-based complexes bearing planar aromatic ligands such as dppz (dipyrido­[3,2-a:2′,3′-c]­phenazine) and tpphz (tetrapyrido­[3,2-a:2′,3′-c:3′′,2′′-h:2′′′,3′′′-j]­phenazine) also intercalate into DNA.^61–64^ These precedents pose the question whether the **1** and **2** assemblies studied in this work can also interact with DNA via this mode, and how this interaction could impact the DNA reactivity in the excited state.

Only spontaneous partial intercalation of **1** was observed in the three 1-μs long MD equilibrium trajectories (Figure S11). The complete intercalation mode of the DIP ligand between DNA consecutive steps has been analyzed through three additional independent MD simulations (∼1 μs per replica) starting from manually pre-intercalated configurations within the minor and major grooves (Figure 6A and 6B, respectively). The distances between the center of masses of the four DNA nucleobases that compose the intercalation site (minor groove) and that of complex **1** clearly show that the complex rapidly departs from the intercalation site within the first few tens of nanoseconds (Figure S12). This finding is consistent with previous reports on minor groove intercalation destabilized by steric hindrance.^60,65–70^

**Figure 6:**
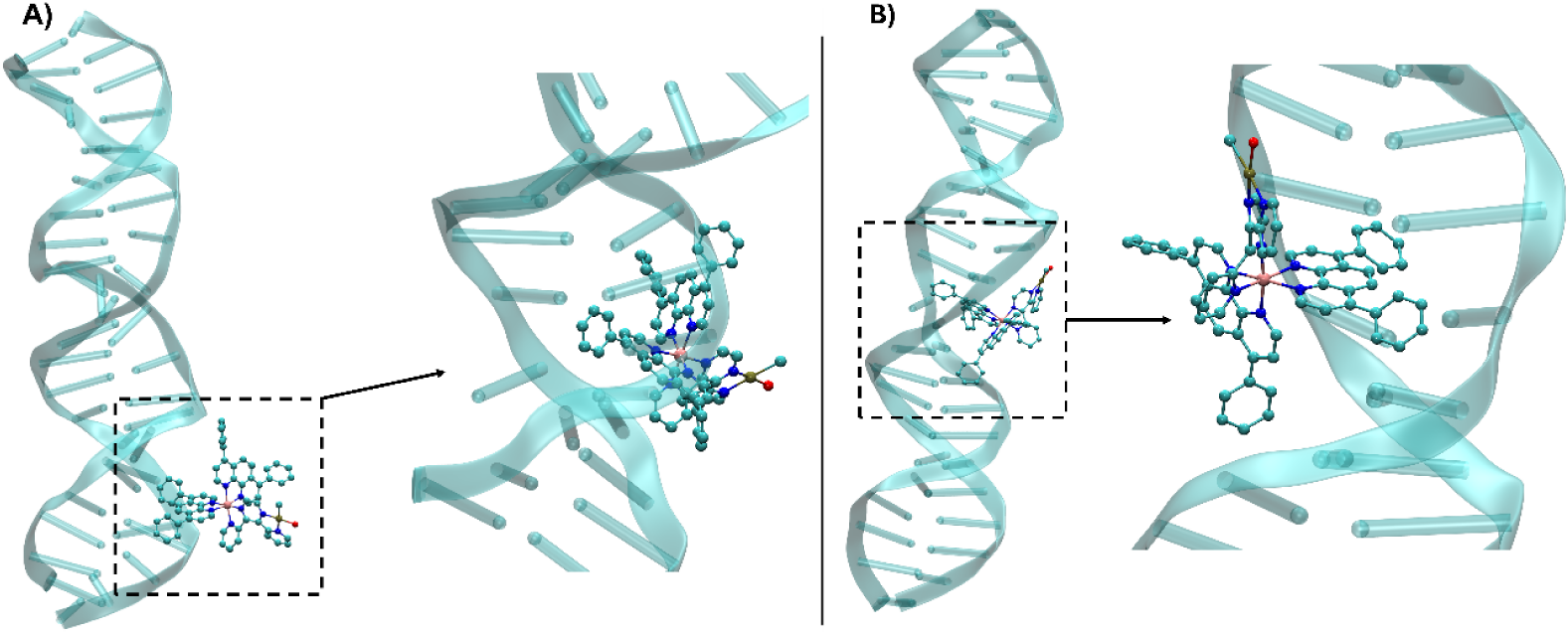
Representative MD snapshots illustrating (A) major-groove and (B) minor-groove intercalation modes of **1** with DNA **A**. Hydrogen atoms omitted for clarity.

In striking contrast, the major-groove intercalated configuration (Figure 6B) is much more persistent and thermodynamically stable, occurring through one phenyl group of the DIP ligand stabilized by effective π-π stacking (Figure 7), as observed in available experiments.^44^ The distance between the center of mass of complex **1** and that of the four nucleobases defining the intercalation site confirms that the ligand remains intercalated throughout the entire simulation time across all replicas (Figure S13). This stability is further supported by an additional simulation using an alternative initial orientation of the complex (Figure S13A), which also remains intercalated over the entire trajectory (Figure S13B). The complex remains intercalated at the end of longer simulation times (2 μs, Figure S14). On the other hand, another 1-μs MD simulation was performed to validate that the mutation in sequence B does not affect the intercalation behavior. No significant differences between sequences A and B were found (Figure S13B). Notably, the simulations also reveal a dynamic exchange between the two phenyl groups, which can further contribute to stabilizing the intercalated state by allowing the adaptation to local DNA fluctuations maintaining the highly favorable stacking interactions (Figure 7).

**Figure 7:**
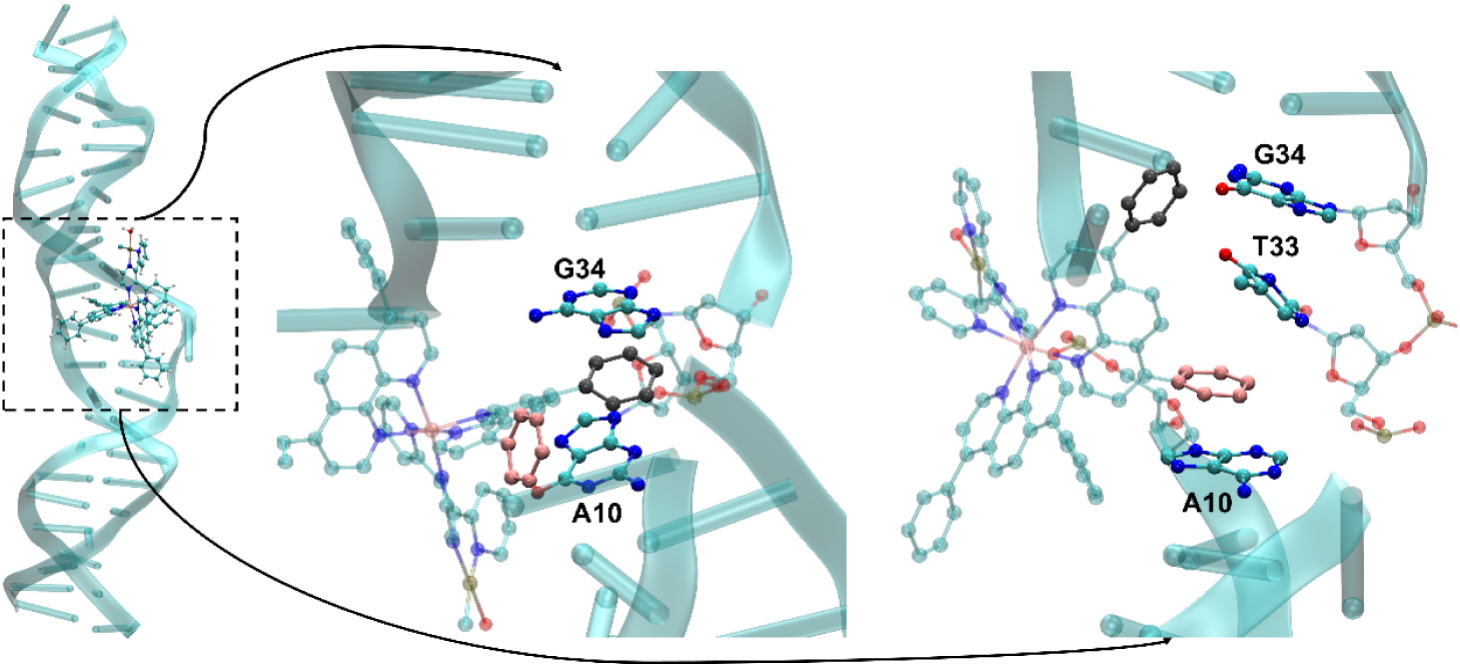
Representative MD snapshots illustrating the π–π stacking interaction between the phenyl ring of complex **1** and the DNA nucleobases. The dynamic exchange between the two phenyl groups is also shown, with the intercalating rings highlighted in gray and pink for clarity.

The impact of this persistent intercalation on DNA structural dynamics is analyzed in the following, since structural deformations can have important biological consequences that could also contribute to the observed phototoxicity of the RuPt assembly in the experiments.^26^ Planar aromatic intercalators such as chloroquine, ethidium bromide (EtBr), and acridine induces DNA bending and, in the case of EtBr, also promotes DNA elongation.^71^ These characteristic deformations, bending and lengthening, have been reported for both organic and metal-containing intercalators.^66,69,72–75^ However, to the best of our knowledge, most computational studies have focused primarily on organic intercalators, possibly due to the complexity of parameterizing force fields for large metal-based systems.

The structural perturbations induced by complex **1** intercalation are elucidated by comparing the dynamics of native DNA with those of the DNA-complex **1** system. The distance between the centers of mass of the terminal nucleotides (Figure 8A) measures elongation, which is increased by approximately 2.8–3.4 Å when **1** is intercalated. Typical increases of around 3.4 Å were reported upon aromatic intercalation.^64,69,71,76^

**Figure 8:**
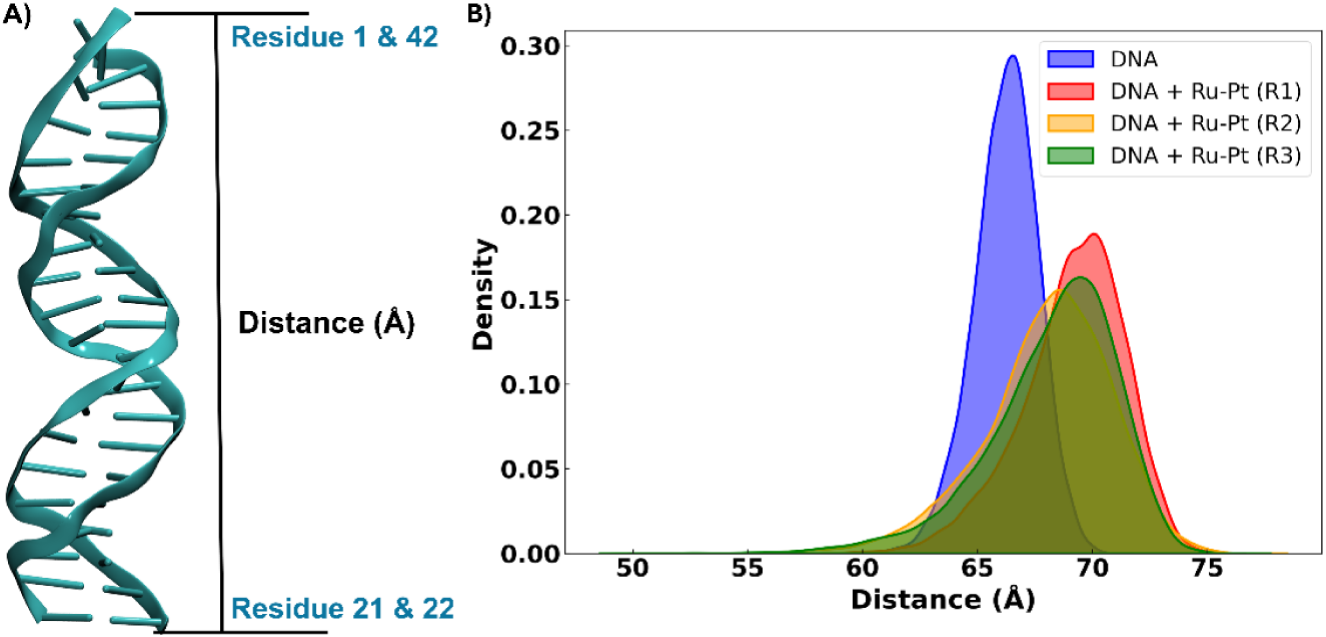
(A) Definition of the double strand length. (B) Histogram of the average DNA length in the absence (DNA) and presence of the complex **1** intercalator throughout three independent replicas (R refers to Replica).

On other hand, the angle defined by the centers of mass of the two terminal base pairs and the central region of the duplex defines the bending deformation (Figure 9A). The average difference between the native DNA and DNA intercalated by **1** ranges from 5° to 15° (Figure 9B), whereas the latter exhibits transient fluctuations reaching angles as small as 105–120° (Figure 9C), inexistent in the reference system. These results clearly reveal the pronounced bending instability induced by intercalation of **1**.

**Figure 9:**
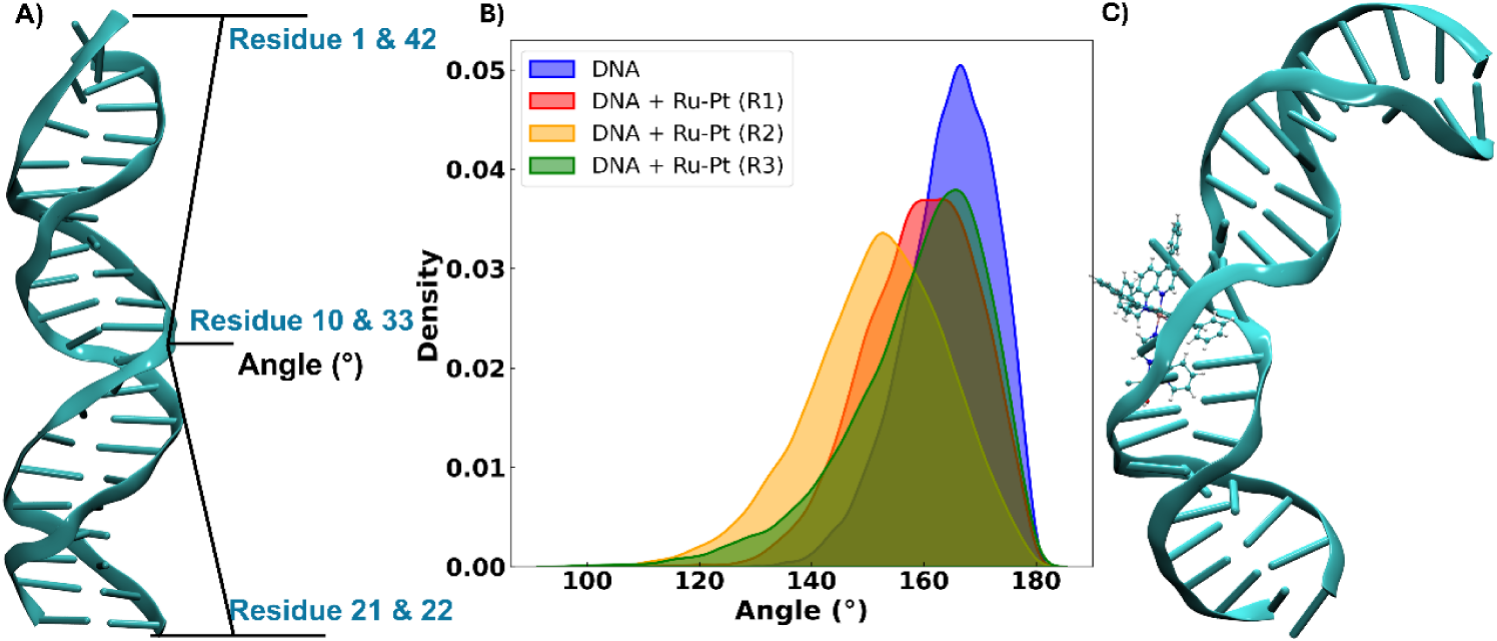
(A) Definition of the bending angle. (B) Bending angles histogram between free and intercalated DNA. (C) Representative snapshot illustrating the bending deformation induced by complex **1** intercalation.

These structural perturbations could correlate with cytotoxicity since they impede the progression of DNA and RNA polymerases,^77^ thereby stalling replication forks and blocking transcription elongation.^67^ Accumulation of these stalled complexes and unresolved lesions can lead to double strand breaks and genome instability, ultimately triggering apoptotic pathways. In essence, the physical deformations induced by intercalation, namely DNA lengthening and bending, act as mechanical roadblocks that convert structural stress into biochemical signals of genomic damage and cell death.^66,67,70,71,74,76,78^ Note that the present work characterizes the structural impact of DNA intercalation although it does not provide direct evidence of these biological consequences. In contrast, the DNA minor/major groove binding modes have a much lighter impact on the double strand, as demonstrated by the analysis of the MD trajectories shown in Figures S15 and S16. Nucleic acid elongation or bending are negligible in these cases, confirming that the pronounced distortions arise exclusively from intercalation.

To further substantiate the intercalative binding of the DIP ligand, binding free energies (Δ*G*) were evaluated to further validate the favorable intercalation of complex **1** within DNA using the MM/PBSA approach. The calculated binding free energy is −8.20 kcal·mol^–1^, indicating a thermodynamically favorable interaction. Decomposition of the binding energy components revealed that the interaction is predominantly driven by van der Waals (VDW) and dispersion forces, consistent with π-π stacking and tight hydrophobic packing within the intercalation site. The VDW contribution is −51.35 kcal·mol^–1^, confirming the dominant role of nonpolar interactions in stabilizing the complex. In contrast, the large gas-phase electrostatic energy (−2367.80 kcal·mol^–1^) is almost completely offset by the polar solvation penalty (+2410.94 kcal·mol^–1^). This almost complete cancellation is typical for charged DNA-ligand systems and represents the expected behavior in MM/PBSA analyses, where the solvent polarization effectively compensates for gas-phase Coulombic attraction.^79,80^ While the binding is predominantly driven by van der Waals interactions, additional stabilization arises from specific hydrogen-bond interactions involving the Pt moiety. Hydrogen-bond analysis reveals significant and persistent interactions with nearby nucleobases, particularly G8 and C9 (Table S6), highlighting the role of the Pt center in reinforcing the overall binding. The Pt fragment is absent in the [Fe­(phen)­(DIP)_2_]^2+^ molecule, which exhibits a lower DNA binding free energy of −7.25 kcal·mol^–1^ (MM-PBSA method, this work) or −5.90 kcal·mol^–1^ (spectrophotometric titration and melting temperature measurements).^44^

Complex **1** intercalates DNA with a comparable or slightly stronger affinity than other known DNA intercalators. Pushkaran et al.^79^ reported binding free energies of −7.6 ± 2.4 kcal·mol^–1^ for the doxorubicin-DNA complex and −5.6 ± 1.4 for the proflavine-DNA intercalation complex, obtained using a computational protocol analogous to the one employed here.^79^ Likewise, ethidium bromide (EtBr) has been found to exhibit binding energies in the range of −6.6 to −7.4 kcal·mol^–1^.^81^

Finally, intercalation does not hinder the photobinding process studied in Figures 4 and 5. On the contrary, it facilitates the Pt-DNA covalent coordination. When complex **1** intercalates into DNA, the Pt center becomes more accessible to the N7 positions of nearby nucleobases, including both guanine and adenine (Figure 10A). The time-dependent distance analysis shows that the platinum center gets close to the N7 atoms of guanine and adenine, frequently sampling distances below 3 Å and maintaining these short separations over extended part of the simulation (Figure 10B). This enhanced and persistent accessibility places the Pt center in a favorable geometric position for nucleophilic attack, thereby increasing the likelihood of photoinduced Pt–N coordination. These results strongly support the appealing idea that intercalation promotes DNA-targeted photoreactions by pre-organizing the complex within the DNA framework, drastically decreasing the available conformational space of the DNA/complex system and the dependence on unspecific diffusion to effectively form the Pt–N bonds.

**Figure 10:**
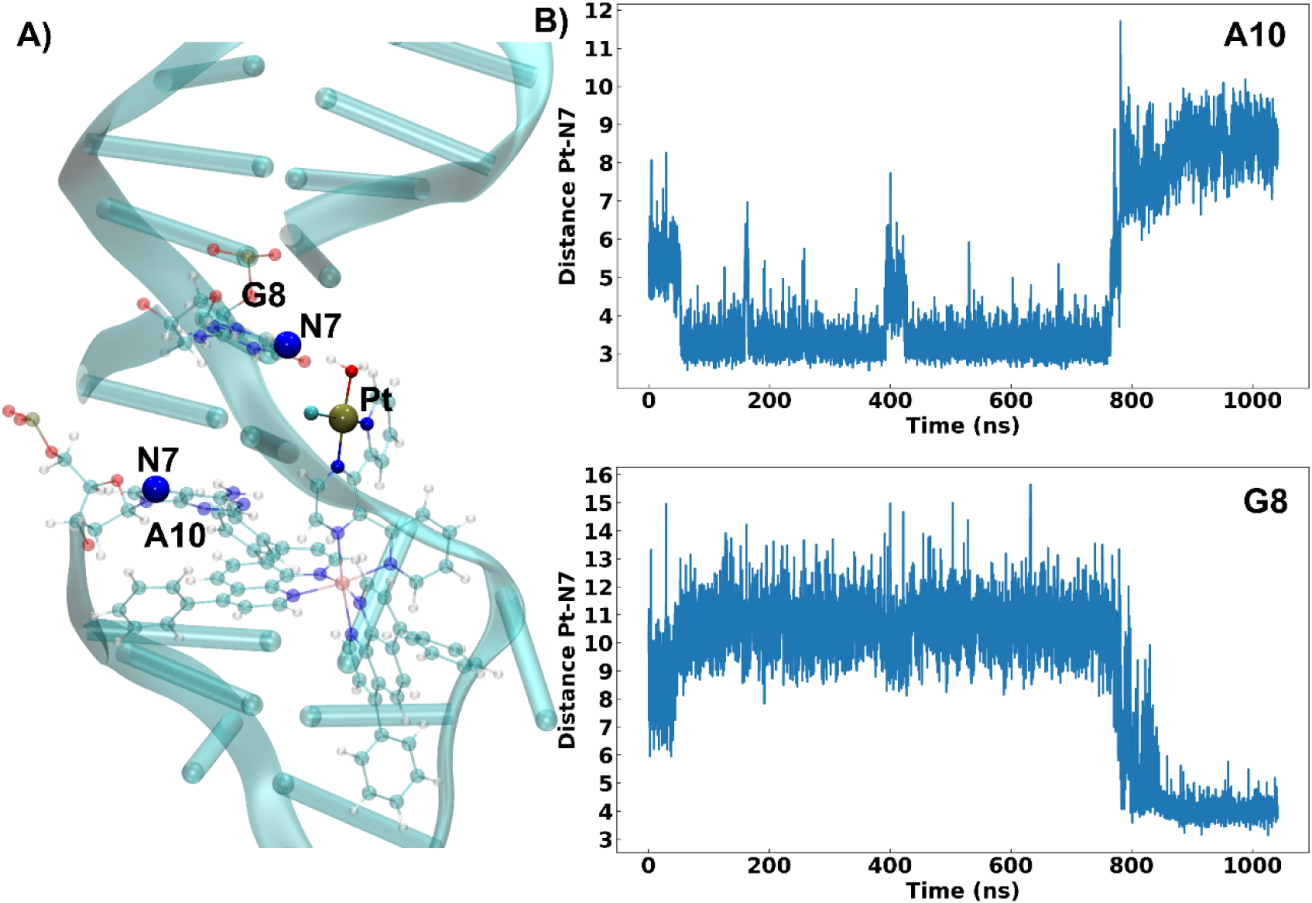
(A) Representative snapshot illustrating the spatial accessibility of the Pt center (CPK brown) and the N7 (CPK blue) of adenine (A10) and guanine (G8). (B) Time evolution of the Pt-N7 for G8 and A10 distances throughout the simulation.

To further quantify this effect, the PMF for Pt-N7 bond formation was computed starting from the intercalated state. The resulting activation barrier (∼5.15 kcal·mol^–1^), which corresponds to an estimated characteristic time of ∼659 ps, is significantly lower than that obtained for the groove-bound configuration (∼6.2 kcal·mol^–1^; ∼3.6 ns) indicating that intercalation provides a faster pathway for covalent binding (Figure 4A). The reaction follows the same concerted mechanism, as illustrated by the representative snapshots shown in Figure S17. Importantly, the intercalation binding mode provides shorter equilibrium distances for the Pt-N7 reaction coordinate. In the triplet state, the DFT/MM free energy well is located at a distance of ∼3.4 Å, significantly shorter than that of the major groove binding mode, whose minimum is at ∼3.9 Å. This is an effect of the environment driven by steric restraints imposed by the intercalation binding mode, which penalizes larger Pt-N7 distances. In the dark, the force field potential reveal stable binding modes at Pt-N7 distances close to ∼3.1 Å. This finding is proved statistically over the 1 microsecond MD simulation, although force field accuracy in describing these interactions is more limited. Globally, it is reasonable to conclude that the sensibly lower free energy barrier and the shorter Pt-N7 distances closer to the transition state (Pt-N7 ∼2.9 Å) will accelerate the photoreaction in the intercalation mode as compared to those of the groove interaction.

DNA intercalation not only reduces the conformational space of the photoreaction and increases its kinetics, but it also benefits the absorption properties of the bimetallic complex because of the π-stacking between nucleobases and the DIP ligand. In particular, the extensive interaction of the aromatic rings with DNA red shifts the MLCT band maximum from 463 nm in aqueous solution to 474 nm, as shown in Figure 11. The spectral change is very modest and characteristic of intercalative binding modes where strong π–π stacking interactions between the aromatic ligand framework and DNA base pairs may stabilize the π* orbitals of the DIP ligand. gld.A similar behavior has been reported for related Ru-polypyridyl complexes^82–84^ and for classical DNA intercalators, including cationic porphyrins, which exhibit bathochromic shifts of approximately 11–16 nm accompanied by significant hypochromicity (45–50%) upon intercalation into DNA.^85^

**Figure 11:**
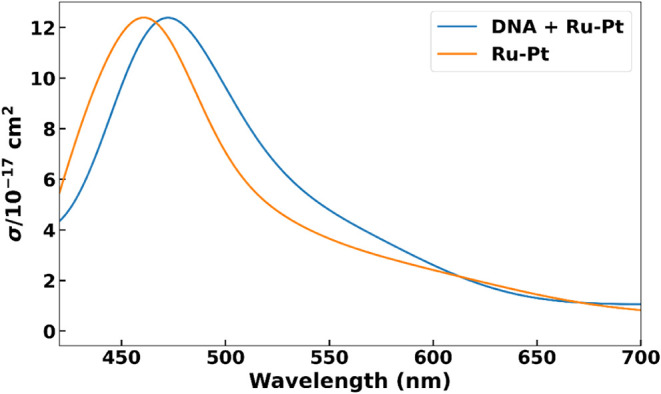
TD-DFT/MM absorption cross sections (σ) of complex **1** (Ru–Pt) intercalated within DNA (blue) and in aqueous solution (orange) computed with 100 independent snapshots extracted from 1 μs MD simulations.

The red shift predicted here, together with the reduced activation barrier for Pt–N bond formation upon intercalation, indicates that DNA intercalation can enhance the efficiency of photoinduced reactions with DNA. This work explores the intra-strand cross-link formation mediated by the nucleophilic attack of purine nucleobases to the electrophile platinum atom, although this benefit may be extended to other photoreactions that target DNA covalently.

## Conclusions and Perspectives

This work establishes a complete theoretical basis for the DNA intra-strand cross link formation of a photoactivated dual PDT/PCT agent that combines a Ru­(II) with a Pt­(II) center. The N7 position of guanine, which is among the most reactive DNA sites, is able to displace the water ligands of the hydrolyzed complexes through a concerted mechanism, forming the corresponding DNA cyclometalated adducts. The systematic characterization of the free energy landscape for the reaction coordinates through multiscale biased QM/MM simulations reveal maximum energy penalties of around ∼6 kcal·mol^–1^ in the triplet excited state, clearly explaining the faster kinetics of the photoinduced pathway with respect to the ground state reactivity of these metallic complexes toward DNA.

The study of the DNA/drug non-covalent interactions indicates that major/minor groove interaction is the preferred interaction mode. Nonetheless, the DIP ligands, frequent in molecular architectures with medicinal purposes, offer also double strand intercalation modes that reduce the conformational space useful for the DNA reaction, a factor that undoubtedly facilitates covalent photobinding. The full intercalation mode is not spontaneous on the microsecond scale, although it is highly thermodynamically favored through an estimated binding free energy of −8.20 kcal·mol^–1^.

The spatial and energetic resolution of the events encompassed in this work suggests the appealing possibility of using DNA intercalation to maximize covalent photochemistry quantum yields toward DNA double strands. Nevertheless, caution must be taken since nonspecific nucleic acid intercalation correlates with toxicity in the dark, therefore, specific activation strategies must be properly developed to mitigate this effect. This might be offset by lower PS doses thanks to enhanced phototoxicity indices given by the synergy between photoexcitation and strong DNA binding, and by restricting the access to the cell nuclei in the dark. In the case of **RuPt** (Scheme 1), only the light-activated form **1** penetrates the nuclear membrane. Therefore, light not only accelerates DNA photochemistry, but also mitigates undesired biological effects in the dark. Note that these mechanisms are totally oxygen-independent, which represent a strategic advance over classical photodynamic therapy.

## Supplementary Material



## Data Availability

The authors declare no competing financial interest. easyPARM, Multispec, VMD, ORCA 6, and Amber 22 (including AmberTools) are publicly available and free of charge. Gaussian 16 and Terachem are commercially available. The computational methodology section and the Supporting Information provide details to reproduce all computations. MD and QM/MM MD trajectories are available in the Zenodo repository 10.5281/zenodo.19232404.

## Notes

1 Full computational details, total simulation times per window, histograms of interatomic distances, PMF convergence, DNA-Pt distances, other representative MD snapshots, drug/DNA distances, S0 stabilities for the QM/MM spectrum (PDF)
