# Supplementary material for: Intercalation Favors DNA Covalent Photobinding in Photoresponsive Dual PDT/PCT Bimetallic Assemblies
Source: J Chem Inf Model. 2026 May 29;66(12):7154–67. doi: 10.1021/acs.jcim.6c00922 (PMC13292199; doi:10.1021/acs.jcim.6c00922)
Supplement: Supplementary file 1 [file ci6c00922_si_001.pdf]

SUPPORTING INFORMATION FOR:

Intercalation Accelerates DNA Covalent Photobinding in Photoresponsive Dual PDT/PCT Bimetallic Assemblies

Abdelazim M. A. Abdelgawwad,[1] Daniel Roca-Sanjuán,[1] Marta E. Alberto,[2,*] Antonio Francés-Monerris[1,*]

[1] Institut de Ciència Molecular, Universitat de València, P.O. Box 22085, València 46071, Spain. A.F.-M. e-mail: antonio.frances@uv.es

[2] Dipartimento di Chimica e Tecnologie Chimiche, Università della Calabria, Arcavacata di Rende I-87036, Italy. M.E.A. e-mail: marta.alberto@unical.it

FULL COMPUTATIONAL DETAILS

Force field parameters for the metal assemblies

Molecular structures of **1** and **2** were optimized using the hybrid functional DFT/B3LYP in combination with the 6-31G* basis set for all nonmetal atoms. For Ru and Pt, the quasi-relativistic Stuttgart-Dresden (SDD)[1] pseudopotential was employed to account for relativistic effects. Analytical frequency calculations were performed at the same level of theory to ensure the absence of imaginary frequencies and to obtain the Hessian matrix required for the force-field parameterization through the easyPARM tool.[2,3]

Atomic partial charges were derived using the restrained electrostatic potential (RESP) method, as implemented in Ambertools.[4] The electrostatic potential was computed at the B3LYP/6-31G* level for all atoms, while the SDD pseudopotential was used for metal centers. All quantum mechanical (QM) calculations were performed using the Gaussian 16 software package without any symmetry restraint.[5]

Classical Molecular Dynamics

The initial structure of the B-DNA double helix with the sequence shown in Figure 1A was generated using the NAB utility of the AmberTools software package.[4] This sequence was chosen as a DNA double helix representative model based on previous literature.[6,7] Figure 1B represents a slight sequence modification at position 10 to facilitate the full formation of a DNA intrastrand cross-link (see below).

Initially, complex **1** was randomly positioned around DNA proximity, and the entire system was solvated in a truncated octahedral box of TIP3P water molecules, ensuring a minimum distance of 10 Å between the solute and the box boundary. System neutrality was achieved by adding 37 Na^+ counterions. The DNA was described using the

parm99 force field[8] with bsc1 corrections,[9] while parameters for the metal complex were generated with the easyPARM tool.[2,3]

The DNA- **1** complex was subjected to energy minimization consisting of 6000 steps of the steepest descent algorithm followed by 6000 steps of the conjugate gradient method. The system was gradually heated to 310.15 K over 200 ps under the NVT ensemble. Production dynamics were then performed under NPT conditions in three independent replicas, each lasting 1 μs. To investigate the intercalation behavior of the complex, an additional set of simulations was carried out by manually positioning the complex between two adjacent DNA base pairs, followed by three replicas of 1 μs each using the same simulation protocol. Additionally, 1-μs simulation was carried out using an alternative initial orientation of complex **1**. To further assess long-timescale stability, one replica was extended by an additional 1-μs, resulting in a total simulation time of 2-μs. Coordinates and velocities were saved every 40 ps. Pressure was maintained at 1 atm using the Monte Carlo barostat, while temperature (310.15 K) was controlled via Langevin dynamics. All simulations employed periodic boundary conditions, with long-range electrostatics treated using the particle mesh Ewald (PME) method and a 10.0 Å nonbonded cutoff. The SHAKE algorithm was applied to constrain bonds involving hydrogen atoms, allowing for a 2-fs integration time step.

All molecular dynamics (MD) simulations were performed using the Amber 22 software package.[10] Trajectories were analyzed with the MDanalysis[11] and visualized through VMD.[12]

Binding Free Energy Calculations

Intercalation free energy for complex **1** was estimated using the molecular mechanics Poisson-Boltzmann surface area (MM-PBSA) approach,[13] which offers reasonable accuracy at a low computational cost by combining molecular mechanics energies with

continuum solvation models. To account for conformational variability, 20 independent replicas of 5 ns each were performed, providing a total of 100 ns of sampling per system. Coordinates were stored every 5 ps, yielding 1000 frames per replica. The first ns of each trajectory was discarded to allow for equilibration, and frames were sampled every 4 ps, resulting in a total of 200 frames × 20 replicas for the MM-PBSA analysis. To assess the suitability of the MM-PBSA protocol for the present intercalative complex **1**, the protocol was first evaluated using a reference system with experimentally characterized binding behavior. The complex $[Fe(phen)(DIP)_2]^{2+}$, which exhibits well-established DNA intercalation and shares structural similarity with the Ru-based fragment of complex **1**, was selected for this purpose.[14] The experimentally reported binding free energy for this complex is -24.7 kJ·mol^{-1} (-5.90 kcal·mol^{-1}). Within this evaluation, a solute dielectric constant of 0.85 was found to provide the closest agreement between the calculated and experimental binding free energies for the reference system. Using this value, the MM-PBSA calculation yielded a binding free energy of -7.25 kcal·mol^{-1}, corresponding to an acceptable deviation of 1.35 kcal·mol^{-1} from the experimental reference. Following this validation step, the same MM-PBSA protocol was applied to the intercalator complex **1**. A solvent probe radius of 1.6 Å and an ionic strength of 150 mM were used in all calculations, while all remaining parameters were maintained at their default values.

QM/MM Simulations and DNA intrastrand cross-link reactivity

Representative snapshots capturing the major groove and intercalation interactions between complex **1** and DNA were extracted from the MD simulations for QM/MM reactivity. For complex **2**, the snapshots extracted for **1** were simply converted to **2** by replacing the Ru center by an Os atom. QM/MM reactivity was studied using the Amber 22/ORCA 6.1[15] interface with the QM-MM electrostatic embedding.[10] In this framework, ORCA computed the QM energies and gradients, while Amber evaluated the

classical MM energies, QM-MM interaction terms (within a 9.0 Å cutoff) and propagated the system dynamics solving the Newton equations of motion with a 2 fs time step. The QM/MM simulations employed periodic boundary conditions and the PME method for long-range electrostatics, with a 10.0 Å nonbonded cutoff. The pressure was maintained at 1 atm using the Monte Carlo barostat (time constant 1 ps), and the temperature was controlled at 310.15 K using Langevin dynamics with a collision frequency of $\gamma = 1\ ps^{-1}$. The SHAKE algorithm was applied to constrain hydrogen bond distances in both QM and MM regions.

For consistency with a previous work,[16] the QM region of the mono-adduct (Figure 2) was treated using the M06 functional combined with the 6-31G basis set for all atoms except Os, Ru, and Pt, which were modeled using the SDD basis set. The ground state (S_0) was treated with restricted DFT/M06, while the triplet state (T_1) was treated using the unrestricted UDFT/M06 method. The QM partition (Figure 2) comprised the metal complex and the interacting nucleobase, with the QM/MM boundary defined across the nucleobase sugar covalent bond. Boundary bonds were capped by hydrogen atoms using the link atom approach as implemented in Amber, in which hydrogen link atoms are placed along the cut bond vector to saturate the QM valence. The charge of the MM atom at the boundary is redistributed among the neighboring MM atoms to avoid over-polarization of the QM region

The system underwent minimization with 250 steps of the steepest descent algorithm, followed by 250 steps of the conjugate gradient algorithm. As a preamble of the umbrella sampling biasing method,[17] a QM/MM relaxed potential energy scan was performed along the Pt-N7 (Guanine 34) distance in the DNA major groove for the triplet state (T_1), using a step size of 0.1 Å from 7.0 to 2.0 Å. The scan was carried out by applying an Amber harmonic restraint with a force constant (k) of 300 $kcal \cdot mol^{-1} \cdot Å^{-2}$. To

avoid hysteresis or direction-dependent effects, the relaxed scan was performed in both forward and backward directions to convergence the potential energy surface along the Pt-N7 distance toward true energy minima.

Initial coordinates for each of the 27 umbrella sampling windows were taken from the bidirectional QM/MM relaxed scans and subsequently minimized through 500 cycles of energy minimization using k = 200 kcal·mol^{-1}·Å^{-2}. Each minimized structure was gradually heated from 0 to 310.15 K over 1.0 ps under the NVT ensemble using k = 60 kcal mol^{-1}·Å^{-2}, followed by 18-25 ps of T_1 excited-state QM/MM production dynamics in the NPT ensemble (see Tables S1 and S2 for the specific simulation times) using the same k = 60 kcal mol^{-1}·Å^{-2}. After ensuring Pt-N7 distance overlap between all simulation windows (Figure S1-S2), the T_1 state potential of mean force (PMF) for the Pt-N7 reaction coordinate was reconstructed using the weighted histogram analysis method (WHAM).[18]

The formation of the second Pt-N covalent bond with an adjacent purine nucleobase leading to the full 1,2-intrastrand crosslink required the easyPARM parameterization[2,3] of the complex **1** coordinated to the N7 position of the first guanine (bi-adduct QM region, Figure 2). On the other hand, since the original DNA sequence shown in Figure 1A does not have any adjacent guanine nucleobases, whose N7 position is deemed as one of the most nucleophilic DNA hotspots,[6,19] the A→C mutation at position 10 was required to complete the intra-strand photoreaction (Figure 1B). The resulting system was subjected to energy minimization consisting of 6000 steps of the steepest descent algorithm followed by 6000 steps of the conjugate gradient method. The system was then gradually heated to 310.15 K over 200 ps under the NVT ensemble, followed by 50 ns of production dynamics performed under the NPT ensemble. A representative snapshot was subsequently extracted with a Pt-N7(Guanine 33) distance of 3.7 Å and used as the starting configuration for QM/MM umbrella sampling. The same

PMF protocol described above was applied, including bidirectional QM/MM relaxed scans, followed by energy minimization, heating, and QM/MM production dynamics of 20-25 ps per window (see Table S3 for details). In this case, a total of 10 windows were required to construct the PMF for formation of the second Pt-N7 bond in which the distances show an excellent overlap between all simulation windows (Figure S3).

QM/MM absorption spectra

To evaluate the effect of DNA intercalation on the electronic absorption spectrum of complex **1**, the UV-Vis spectrum of the DNA/**1** system and of **1** in pure water solution (hereafter, water/**1**) was computed by means of QM/MM methodology. 100 equally spaced representative geometries were extracted from MD simulations. In the DNA/**1** case, snapshots were extracted from the 1 μs-long replica 1, whereas for water/**1** an additional MD simulation was run in which **1** is solvated inside a cubic water box (minimum distance of 10 Å between the solute and the box boundary). This simulation was carried out using the same protocol described previously, including 20,000 steps of energy minimization, 200 ps of equilibration, followed by a 200 ns production run.

The S_0 state of each snapshot was equilibrated during 100 steps using the Amber/Terachem interface.[20–22] The QM region of DNA/**1** included the metal complex and the four nucleobases surrounding the intercalation site, namely Adenine 10 and 11 and Thymine 32 and 33. For water/**1**, the QM region contained only complex **1**. For the sake of consistency, the QM/MM equilibration of both DNA/**1** and water/**1** systems was performed using the PBE0 functional with the 6-31G basis set for all atoms and the LANL2DZ basis set for the Pt and Ru metal centers. A QM-MM cutoff of 5 Å was applied to generate the electrostatic point charges from the surrounding MM region.

The Amber/Gaussian 16 interface was used to check for singlet unstable solutions, all instabilities were found to be negligible (Figure S13). The absorption spectrum was

finally computed on top of the QM/MM equilibrated structures making use of the Amber/Gaussian 16 interface and the TD-M06/6-31+G(d,p)/SDD/MM level of theory, a method widely validated in the literature.[16] 100 singlet excited states and associated oscillator strengths were computed vertically on top of each snapshot to ensure the coverage of the visible wavelengths ($\lambda \geq 420$ nm) relevant in phototherapy. Cross sections (σ) were computed using all sets of vertical excitation energies and oscillator strengths with the code MULTISPEC developed in our group.[23]

Table S1. QM/MM simulation times (timestep = 2 fs) of the production runs used to build the PMF for T_1 of complex **1**.

Window (Å)	Simulation time (fs)
2.0	10060
2.2	10161
2.4	10473
2.6	10184
2.8	10159
2.9	10127
3.0	10200
3.2	10200
3.4	10200
3.6	10200
3.8	10200
4.0	10200
4.2	10200
4.4	10200
4.6	10200
4.8	9770
5.0	10200
5.2	10025
5.4	10200
5.6	10200
5.8	10200
6.0	10200
6.2	10200
6.4	10200
6.6	10469
6.8	10200
7.0	10200
**Total**	**275028**

Table S2. QM/MM simulation times (timestep = 2 fs) of the production runs used to build the PMF for S_0 of complex **1**.

Window (Å)	Simulation time (fs)
2.00	12750
2.20	12750
2.40	9850
2.50	12750
2.60	10200
2.80	10200
3.00	10200
3.20	10200
3.40	10200
3.60	8955
3.80	10200
4.00	10200
4.20	10200
4.40	10200
4.60	12391
4.80	12393
5.00	12394
5.20	12531
5.40	12300
5.60	12176
5.80	11294
6.00	11258
6.20	11262
6.40	11328
6.60	10200
6.80	11194
7.00	12750
**Total**	**302326**

Table S3. QM/MM simulation times (timestep = 2 fs) of the production runs used to build the PMF for T_1 of complex **2**.

Window (Å)	Simulation time (fs)
2.0	10950
2.2	11142
2.4	11254
2.6	11158
2.8	9904
2.9	11524
3.0	10200
3.2	10200
3.4	10200
3.6	10200
3.8	10200
4.0	10200
4.2	10200
4.4	10200
4.6	12692
4.8	12428
5.0	12370
5.2	12673
5.4	12700
5.6	12333
5.8	11399
6.0	11567
6.2	11542
6.4	11541
6.6	11550
6.8	11428
7.0	11347
**Total**	**303102**

Table S4. QM/MM simulation times (timestep = 2 fs) of the production runs used to build the PMF for T_1 of complex 1 with N7 of guanine.

Window (Å)	Simulation time (fs)
2.00	10200
2.20	10200
2.40	10200
2.60	10512
2.70	13024
2.80	12750
2.90	16320
3.00	11684
3.20	10733
3.40	10200
**Total**	**115823**

Table S5. QM/MM simulation times (timestep = 2 fs) of the production runs used to build the PMF for T_1 of complex 1 with N7 of guanine, starting from the intercalated configuration.

Window (Å)	Simulation time (fs)
2.00	10200
2.20	10200
2.40	9644
2.60	10200
2.80	10685
2.90	11382
3.00	11032
3.20	10200
3.40	12750
3.60	13487
3.80	10549
4.00	12750
4.20	10200
**Total**	**143279**

Table S6. Highest hydrogen-bond occupancies (%) over time of **1** with different DNA residues. Whole complex refers to hydrogen bonds between DNA and any atom of the bimetallic complex, whereas Pt moiety refers to the participation of the Pt center and its coordination sphere (coordinating nitrogen atoms, water molecule, and chloride atom). A 100% hydrogen bond occupancy implies that a H bond is formed in all simulation frames. The thresholds for interatomic distance and D-H···A angle (D= donor, A= acceptor) to consider H bonding are ≤3 Å and ≥ 150º, respectively.

DNA Residue	Whole complex	Exclusive to the Pt moiety
G8	44.17	43.43
C9	26.02	17.41
A10	16.27	1.02
T33	5.46	
G34	14.73	

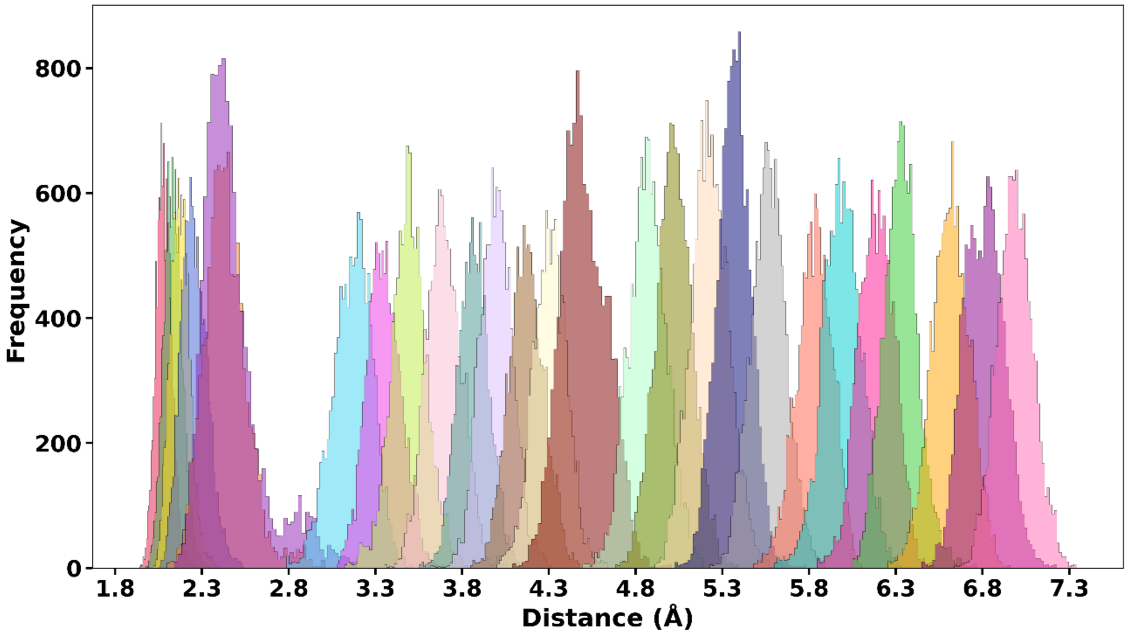

Figure S1. Histogram of Pt-N7 distances (Å) for complex 1 in the triplet excited state (T_1), obtained from the 27 umbrella sampling windows used in the PMF calculation. Data was collected with a sampling frequency of 1 fs per step, illustrating the distribution of configurations sampled along the reaction coordinate.

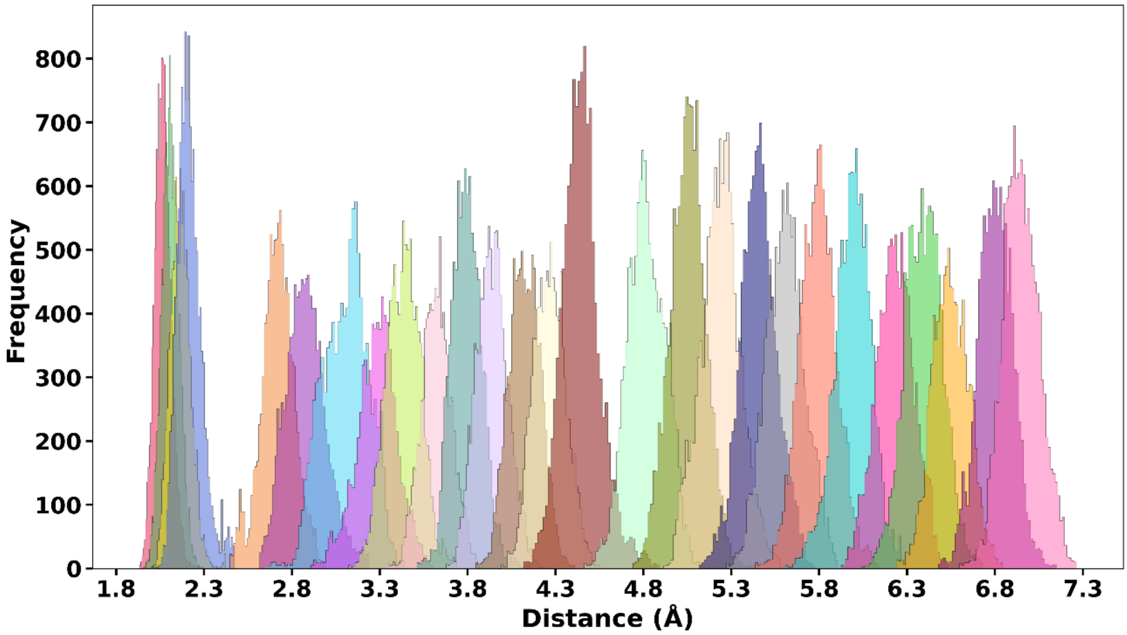

Figure S2. Histogram of Pt-N7 distances (Å) for complex 1 in the ground state (S0), obtained from the 27 umbrella sampling windows used in the PMF calculation. Data was collected with a sampling frequency of 1 fs per step, illustrating the distribution of configurations sampled along the reaction coordinate.

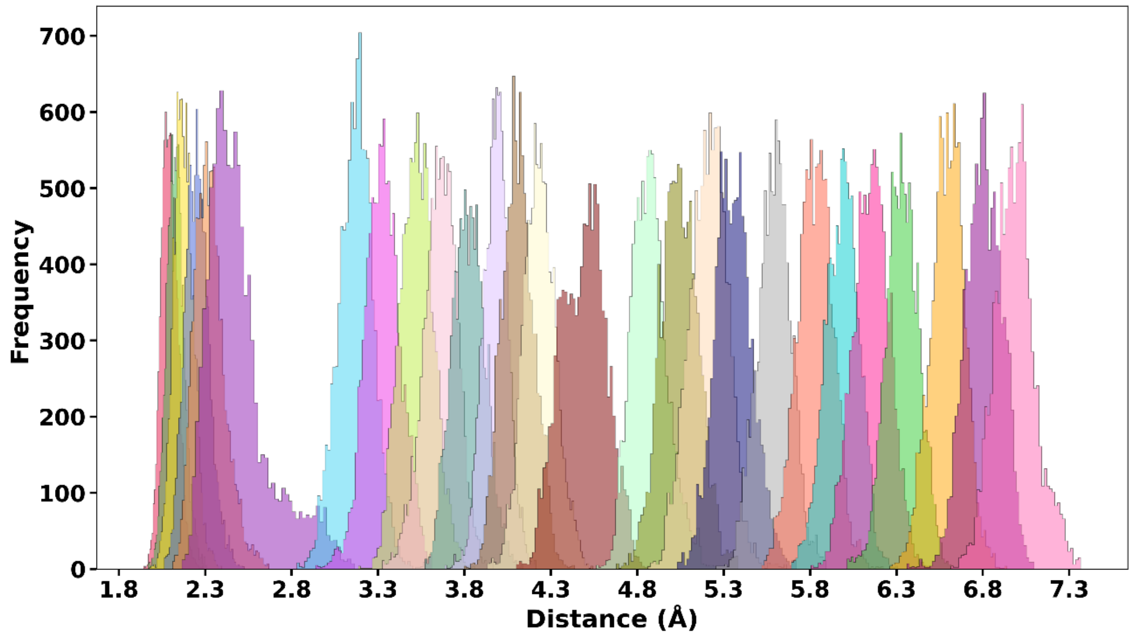

Figure S3. Histogram of Pt-N7 distances (Å) for complex 2 in the excited state (T_1), obtained from the 27 umbrella sampling windows used in the PMF calculation. Data was collected with a sampling frequency of 1 fs per step, illustrating the distribution of configurations sampled along the reaction coordinate.

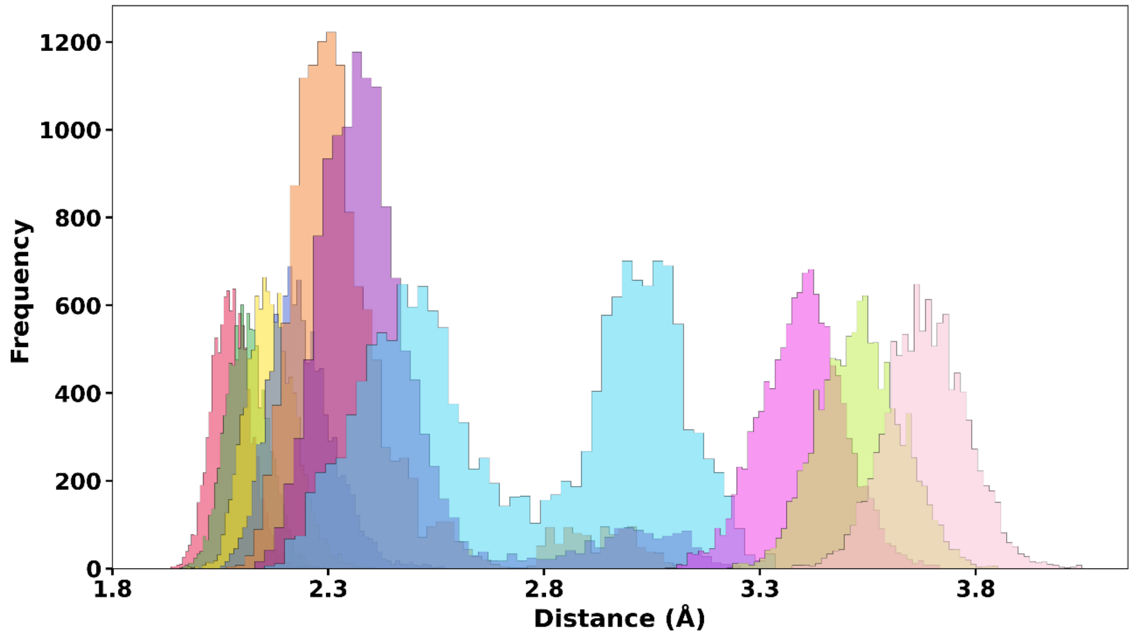

Figure S4. Histogram of the second covalent bond Pt-N7 distances (Å) for the complex 1 in the excited state (T_1), obtained from the 10 umbrella sampling windows used in the PMF calculation. Data was collected with a sampling frequency of 1 fs per step, illustrating the distribution of configurations sampled along the reaction coordinate.

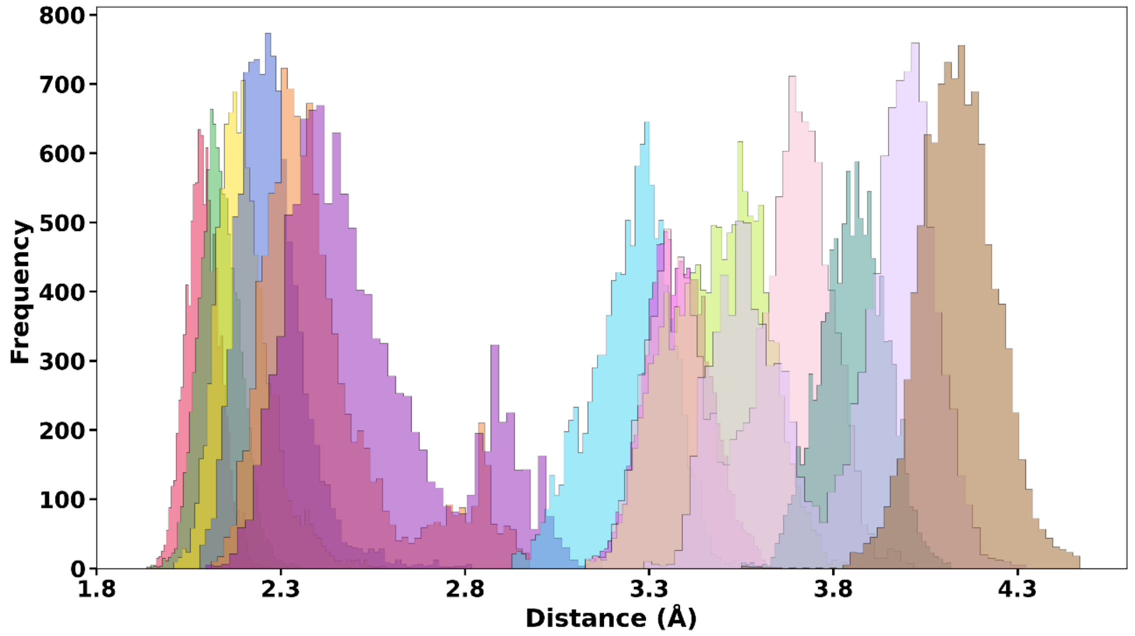

Figure S5. Histogram of the covalent bond Pt-N7 distances (Å) for the complex 1 in the excited state (T_1), starting from the intercalated configuration, obtained from the 9 umbrella sampling windows used in the PMF calculation. Data was collected with a sampling frequency of 1 fs per step, illustrating the distribution of configurations sampled along the reaction coordinate.

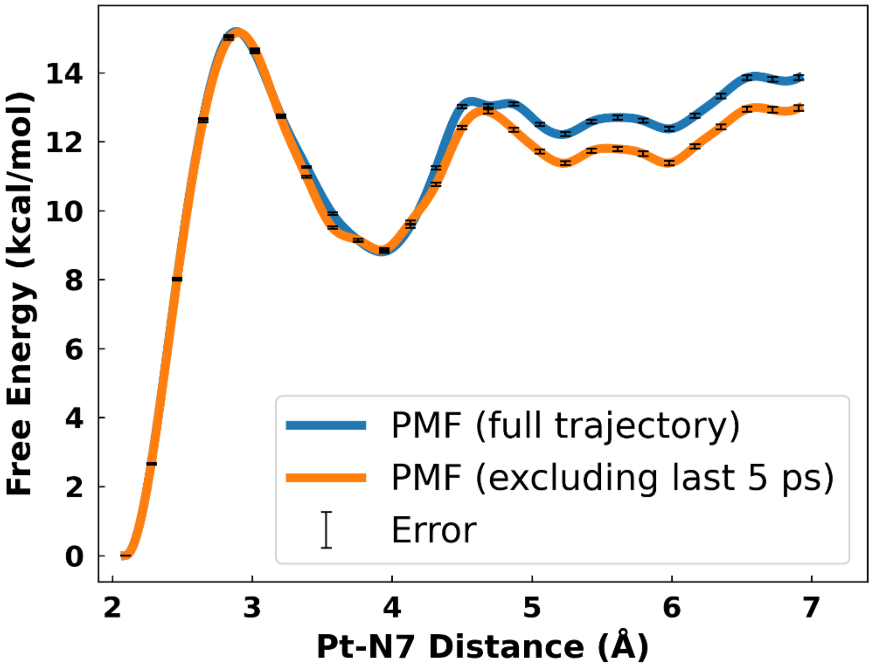

Figure S6. PMF convergence analysis along the Pt-N7 distance. Comparison between the full trajectory (blue) and PMF excluding the last 5 ps per window (orange) shows good agreement in the barrier region (2.0-4.0 Å), indicating satisfactory convergence.

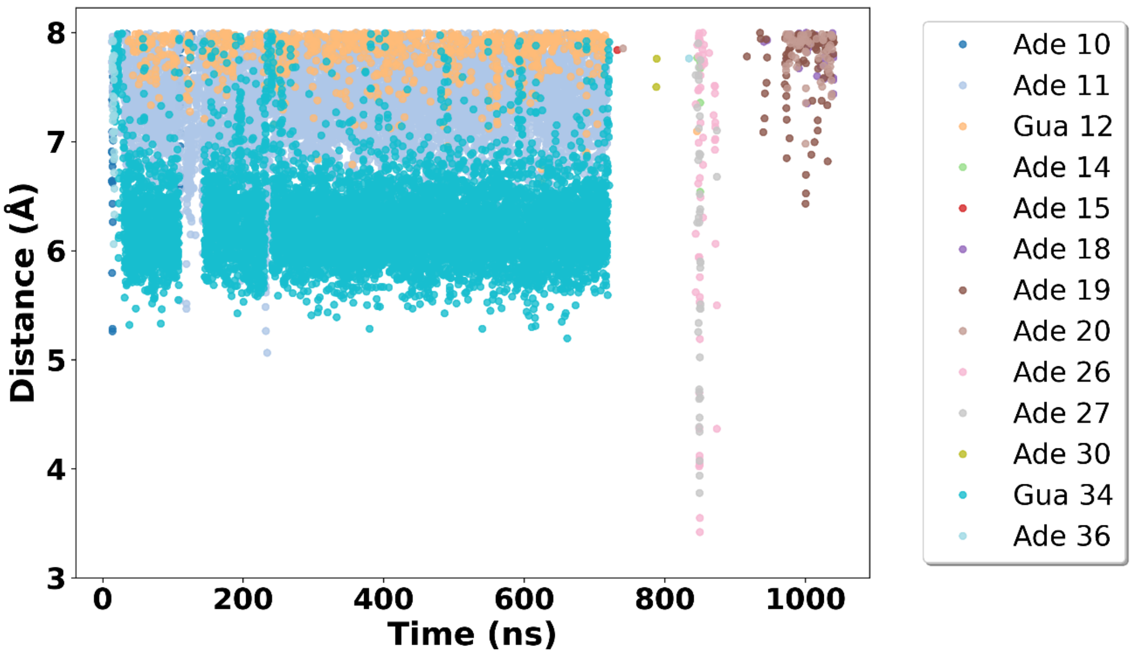

Figure S7. Computed distances between the Pt center of complex 1 and N7 positions of all purine nucleobases throughout the 1000 ns MD simulation of replica 1.

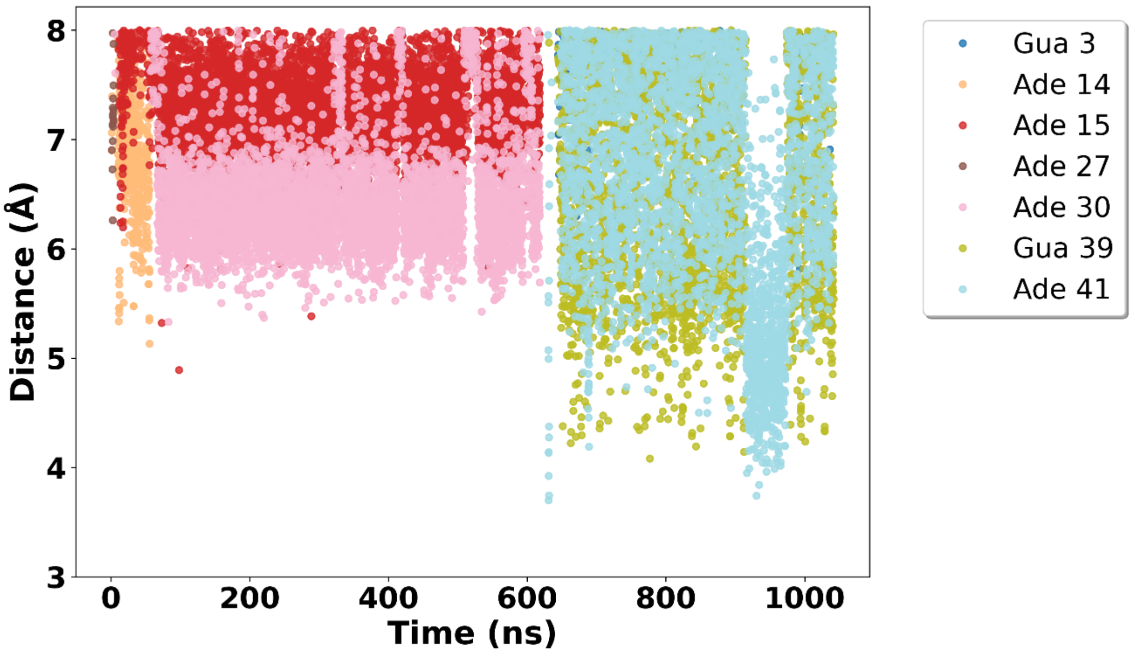

Figure S8. Computed distances between the Pt center of complex 1 and N7 positions of all purine nucleobases throughout the 1000 ns MD simulation of replica 2.

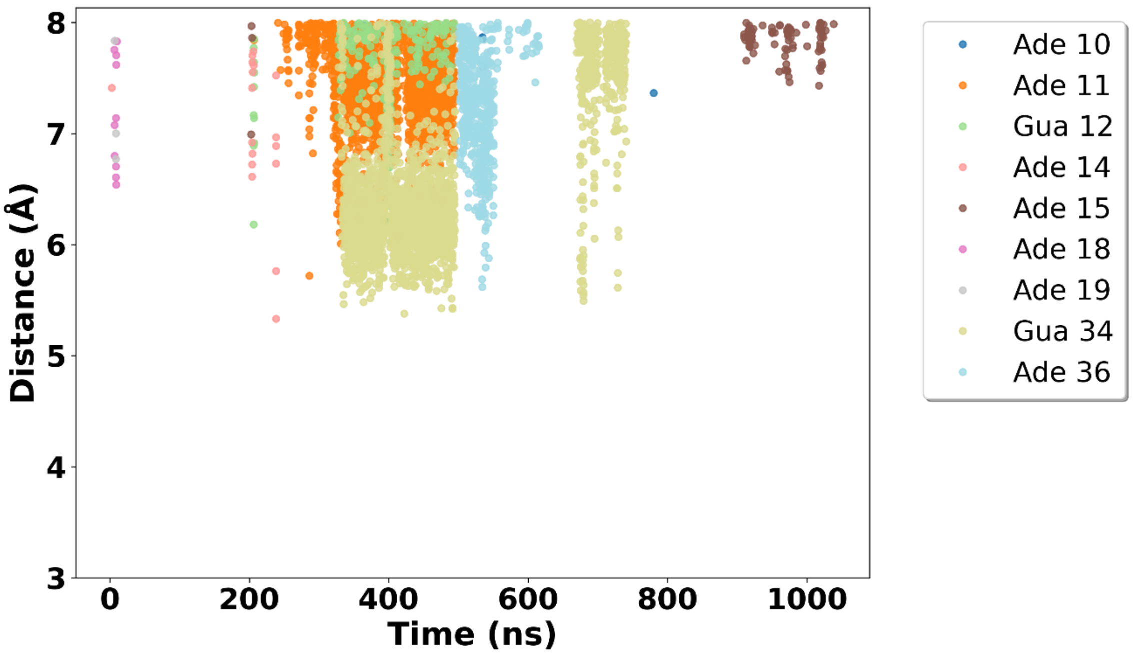

Figure S9. Computed distances between the Pt center of complex 1 and N7 positions of all purine nucleobases throughout the 1000 ns MD simulation of replica 3.

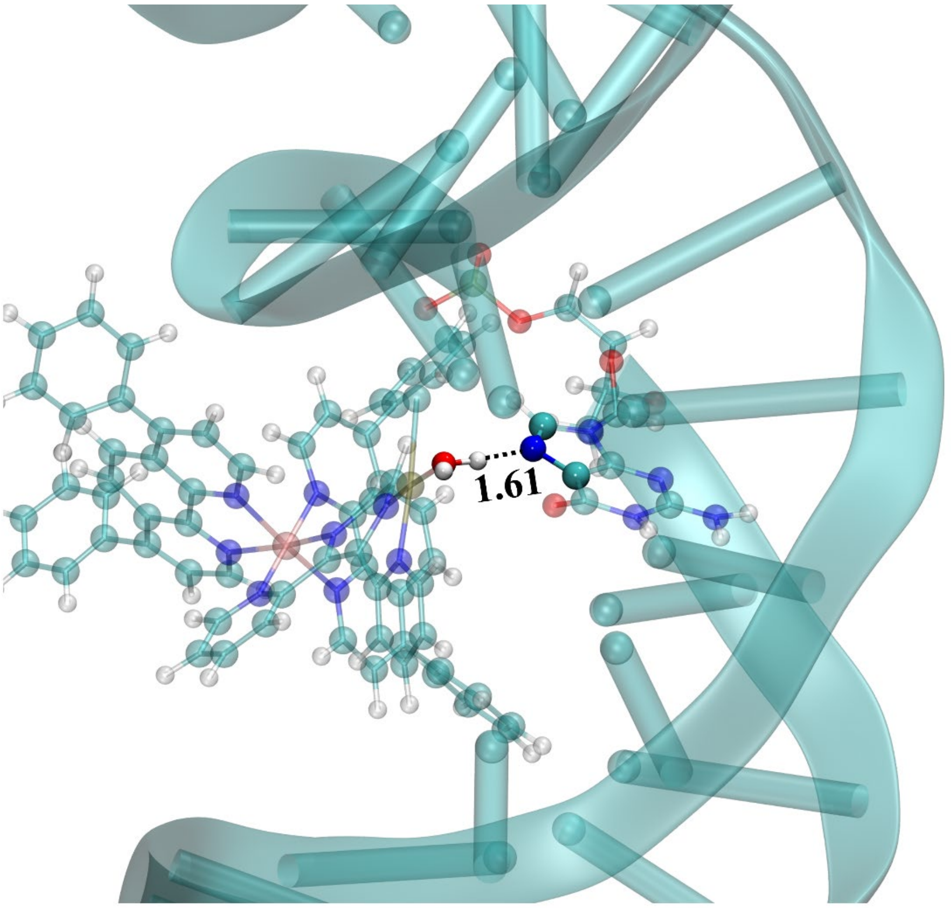

Figure S10. Hydrogen bonding between the water hydrogen (Hw) and the N7 atom of guanine in the **1** + **A** reaction.

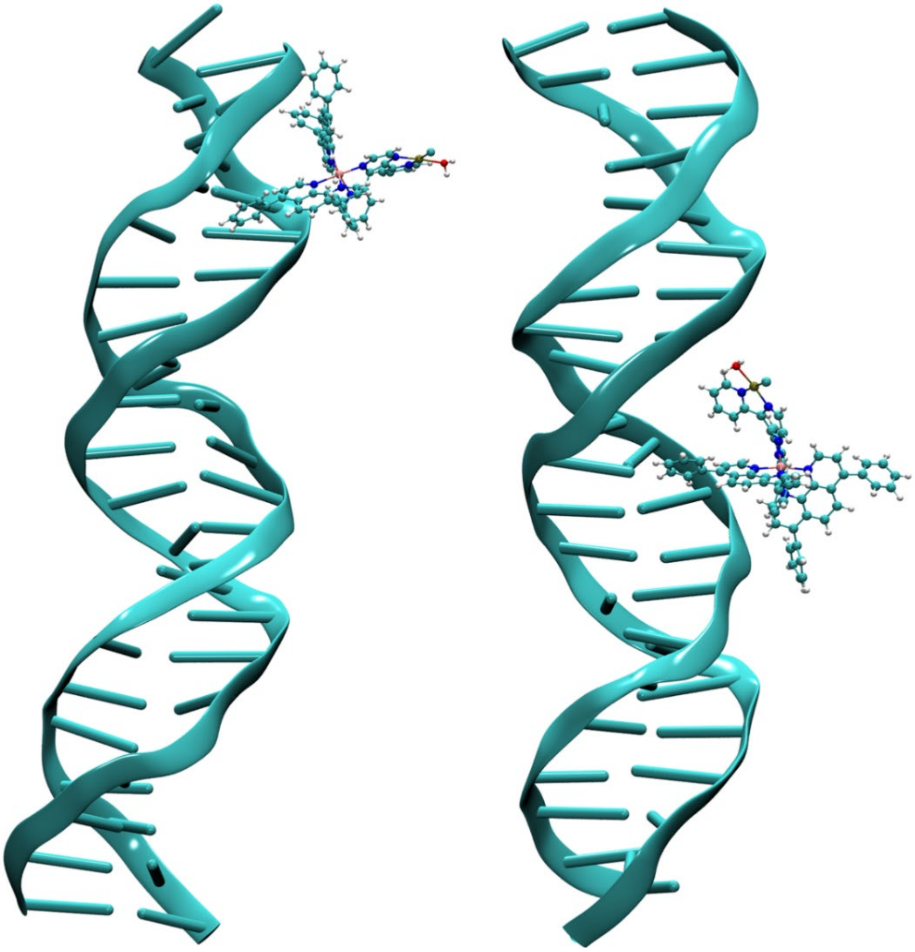

Figure S11. Representative MD snapshot depicting the intercalation tendency between complex **1** and DNA.

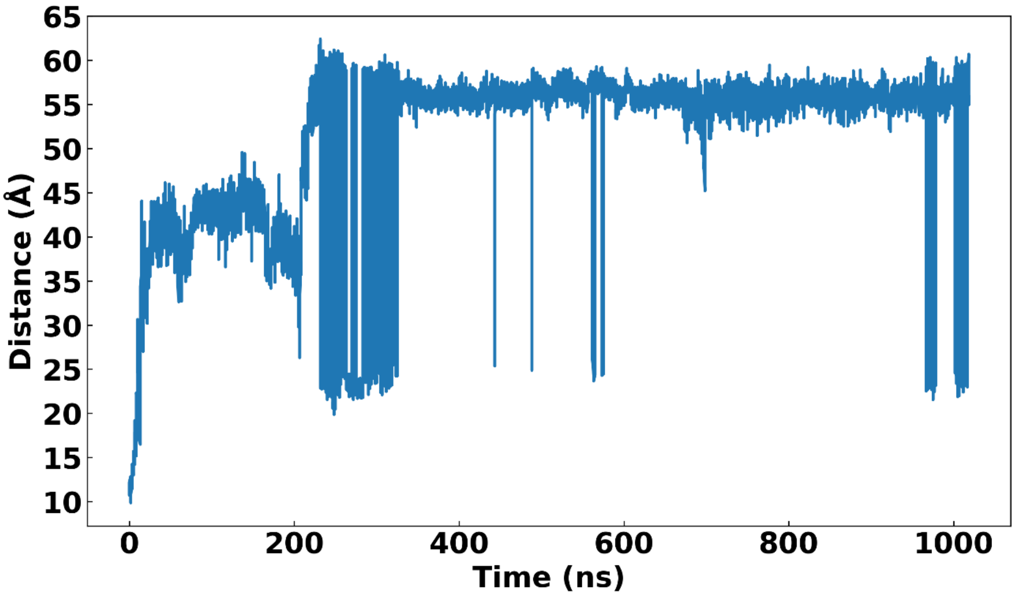

Figure S12. Minor groove intercalation of **1**. Computed distance between the center of mass of complex 1 and center of mass of residues (A6, A5, T37, T38) of DNA throughout the 1000 ns MD simulation. A and T refer to Adenine and Thymine respectively.

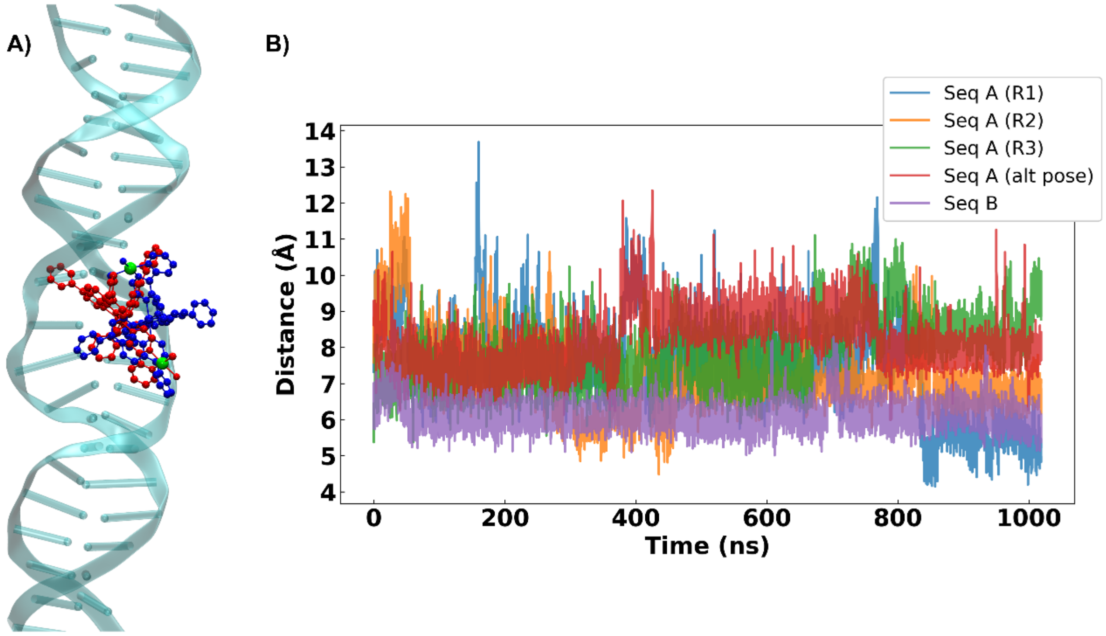

Figure S13. Major groove intercalation of **1**. (A) Representative orientations of complex **1** in the DNA major groove. The Pt atom is highlighted in green to emphasize differences in ligand orientation. (B) Time evolution of the distance between the center of mass of complex **1** and the center of mass of DNA residues (A10, A11, T32, T33) over 1000 ns molecular dynamics simulations. Three independent replicas starting from the same initial conformation were performed for sequence A (R1-R3), along with an additional simulation initialized from an alternative ligand orientation (alt pose). One simulation was performed for sequence B. A and T denote adenine and thymine, respectively.

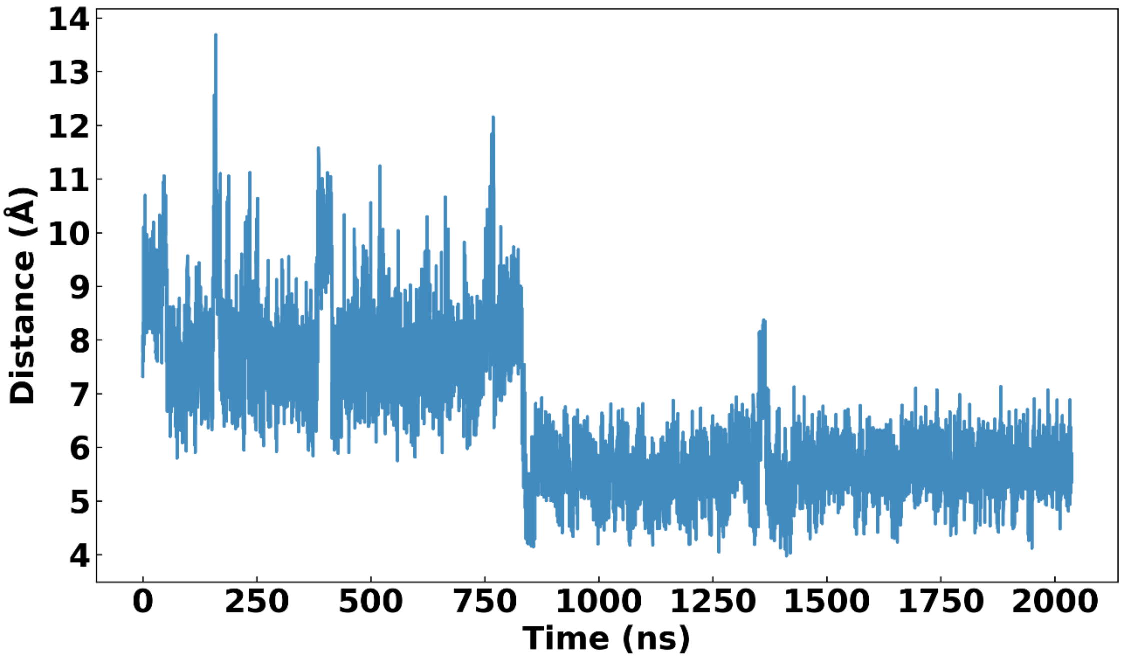

Figure S14. Major-groove intercalation of complex 1 with extended simulation time (2000 ns). Time evolution of the distance between the center of mass of complex 1 and that of the DNA residues (A10, A11, T32, T33) during a 1000 ns molecular dynamics simulation.

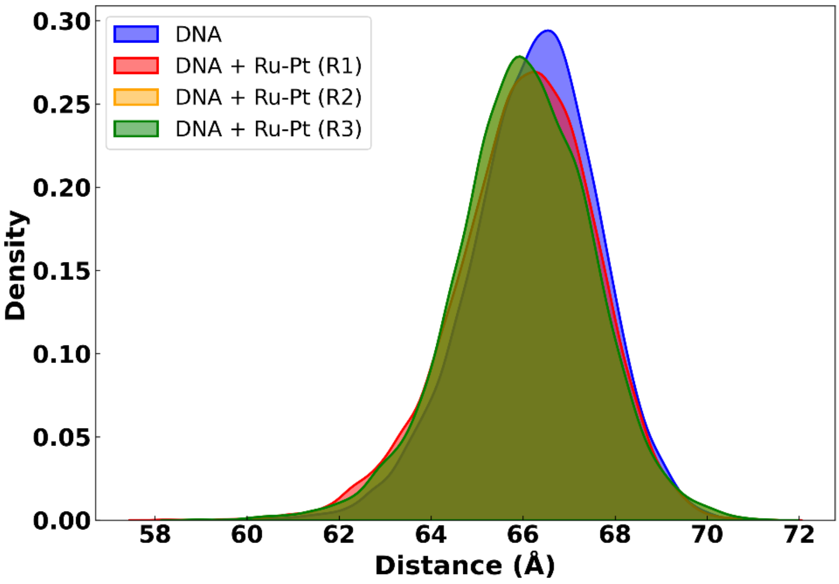

Figure S15. Comparison of the computed distance between free and Ru-Pt-DNA complex.

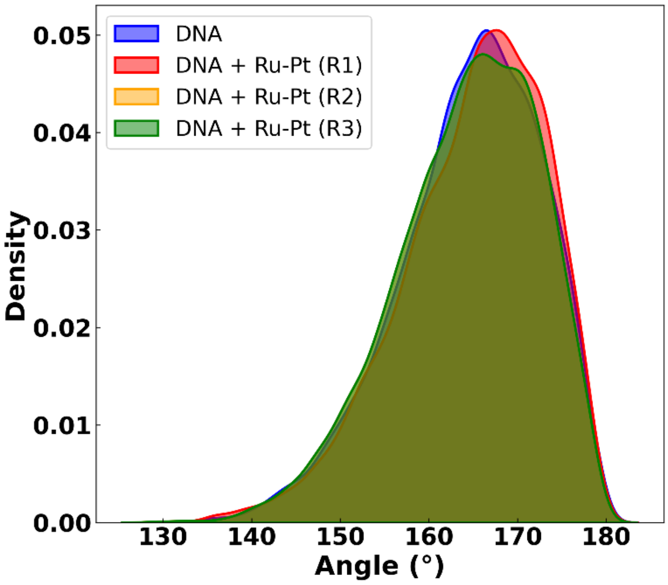

Figure S16. Comparison of the computed angle bending between free and Ru-Pt-DNA complex.

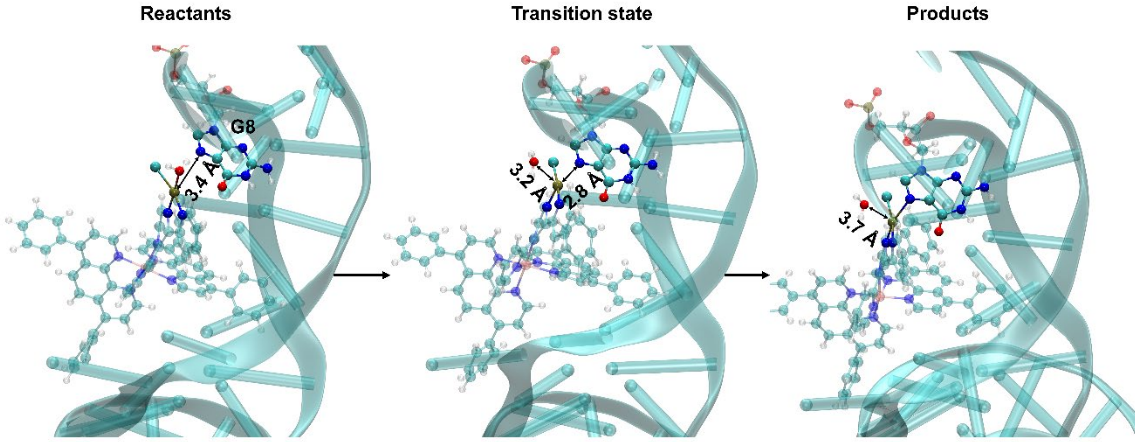

Figure S17. Representative QM/MM snapshots extracted from umbrella sampling windows, starting from the intercalated configuration and illustrating the formation of the Pt–N7 bond.

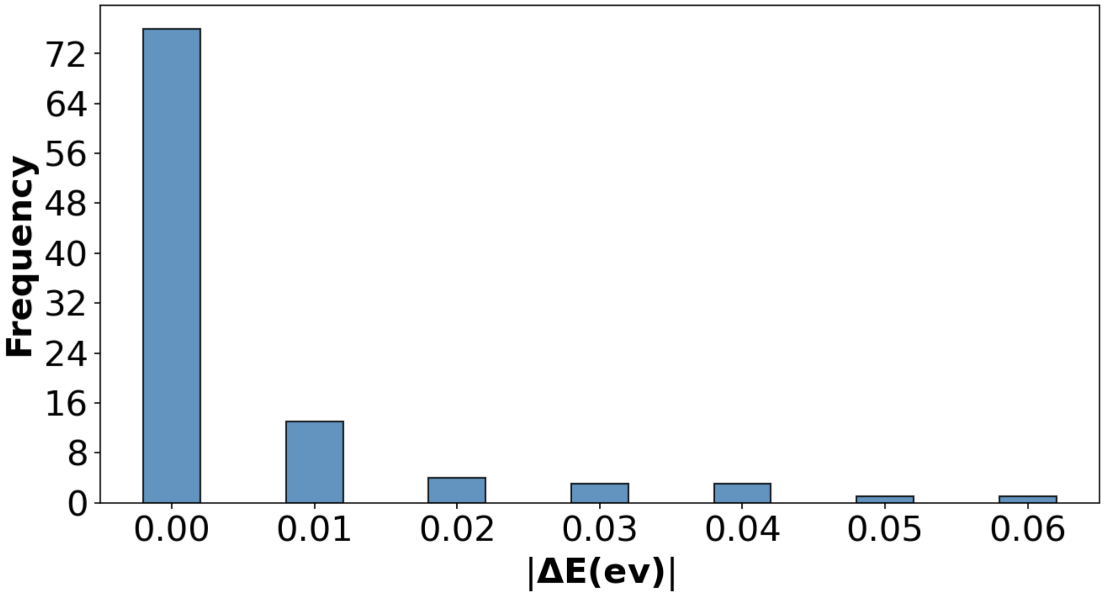

Figure S18. Histograms of the energy difference between the closed-shell and the unrestricted solutions of the S_0 state for the 100 snapshots used to compute the UV-Vis spectrum of **1** intercalated inside the DNA strand. Stability is computed with the Gaussian 16 keyword opt=stable. $\Delta E = 0$ implies that the closed-shell solution is already stable. In all cases, ΔE is negligible.

REFERENCES

(1) Andrae, D.; Häußermann, U.; Dolg, M.; Stoll, H.; Preuß, H. Energy-Adjustedab Initio Pseudopotentials for the Second and Third Row Transition Elements. *Theor. Chim. Acta* **1990**, *77* (2), 123–141. https://doi.org/10.1007/BF01114537.

(2) Abdelgawwad, A. M. A.; Francés-Monerris, A. EasyPARM: Automated, Versatile, and Reliable Force Field Parameters for Metal-Containing Molecules with Unique Labeling of Coordinating Atoms. *J. Chem. Theory Comput.* **2025**, *21* (4), 1817–1830. https://doi.org/10.1021/acs.jctc.4c01272.

(3) Abdelgawwad, A. M. A.; Francés-Monerris, A. EasyPARM v4.00: A Python-Based Tool for the Automated Parameterization of Metalloproteins and Metal-Organic Polyhedra with Multiple Metal Centers. *J. Chem. Phys.* **2025**, *163* (22), 222501. https://doi.org/10.1063/5.0301038.

(4) Case, D. A.; Aktulga, H. M.; Belfon, K.; Cerutti, D. S.; Cisneros, G. A.; Cruzeiro, V. W. D.; Forouzesh, N.; Giese, T. J.; Götz, A. W.; Gohlke, H.; Izadi, S.; Kasavajhala, K.; Kaymak, M. C.; King, E.; Kurtzman, T.; Lee, T.-S.; Li, P.; Liu, J.; Luchko, T.; Luo, R.; Manathunga, M.; Machado, M. R.; Nguyen, H. M.; O'Hearn, K. A.; Onufriev, A. V; Pan, F.; Pantano, S.; Qi, R.; Rahnamoun, A.; Risheh, A.; Schott-Verdugo, S.; Shajan, A.; Swails, J.; Wang, J.; Wei, H.; Wu, X.; Wu, Y.; Zhang, S.; Zhao, S.; Zhu, Q.; Cheatham, T. E. I. I. I.; Roe, D. R.; Roitberg, A.; Simmerling, C.; York, D. M.; Nagan, M. C.; Merz, K. M. Jr. AmberTools. *J. Chem. Inf. Model.* **2023**, *63* (20), 6183–6191. https://doi.org/10.1021/acs.jcim.3c01153.

(5) Frisch, M. J.; Trucks, G. W.; Schlegel, H. B.; Scuseria, G. E.; Robb, M. a.; Cheeseman, J. R.; Scalmani, G.; Barone, V.; Petersson, G. a.; Nakatsuji, H.; Li, X.; Caricato, M.; Marenich, a. V.; Bloino, J.; Janesko, B. G.; Gomperts, R.; Mennucci, B.; Hratchian, H. P.; Ortiz, J. V.; Izmaylov, a. F.; Sonnenberg, J. L.; Williams; Ding, F.; Lipparini, F.; Egidi, F.; Goings, J.; Peng, B.; Petrone, A.; Henderson, T.; Ranasinghe, D.; Zakrzewski, V. G.; Gao, J.; Rega, N.; Zheng, G.; Liang, W.; Hada, M.; Ehara, M.; Toyota, K.; Fukuda, R.; Hasegawa, J.; Ishida, M.; Nakajima, T.; Honda, Y.; Kitao, O.; Nakai, H.; Vreven, T.; Throssell, K.; Montgomery Jr., J. a.; Peralta, J. E.; Ogliaro, F.; Bearpark, M. J.; Heyd, J. J.; Brothers, E. N.; Kudin, K. N.; Staroverov, V. N.; Keith, T. a.; Kobayashi, R.; Normand, J.; Raghavachari, K.; Rendell, a. P.; Burant, J. C.; Iyengar, S. S.; Tomasi, J.; Cossi, M.; Millam, J. M.; Klene, M.; Adamo, C.; Cammi, R.; Ochterski, J. W.; Martin, R. L.; Morokuma, K.; Farkas, O.; Foresman, J. B.; Fox, D. J. G16_C01. 2016, p Gaussian 16, Revision C.01, Gaussian, Inc., Wallin.

(6) Abdelgawwad, A. M. A.; Monari, A.; Tuñón, I.; Francés-Monerris, A. Spatial and Temporal Resolution of the Oxygen-Independent Photoinduced DNA Interstrand Cross-Linking by a Nitroimidazole Derivative. *J. Chem. Inf. Model.* **2022**, *62* (13), 3239–3252. https://doi.org/10.1021/acs.jcim.2c00460.

(7) Han, Y.; Chen, W.; Kuang, Y.; Sun, H.; Wang, Z.; Peng, X. UV-Induced DNA Interstrand Cross-Linking and Direct Strand Breaks from a New Type of Binitroimidazole Analogue. *Chem. Res. Toxicol.* **2015**, *28* (5), 919–926. https://doi.org/10.1021/tx500522r.

(8) Cornell, W. D.; Cieplak, P.; Bayly, C. I.; Gould, I. R.; Merz, K. M.; Ferguson, D. M.; Spellmeyer, D. C.; Fox, T.; Caldwell, J. W.; Kollman, P. A. A Second Generation Force Field for the Simulation of Proteins, Nucleic Acids, and Organic Molecules. *J. Am. Chem. Soc.* **1995**, *117* (19), 5179–5197. https://doi.org/10.1021/ja00124a002.

(9) Ivani, I.; Dans, P. D.; Noy, A.; Pérez, A.; Faustino, I.; Hospital, A.; Walther, J.; Andrio, P.; Goñi, R.; Balaceanu, A.; Portella, G.; Battistini, F.; Gelpí, J. L.; González, C.; Vendruscolo, M.; Laughton, C. A.; Harris, S. A.; Case, D. A.; Orozco, M. PARMBSC1: A Refined Force-Field for DNA Simulations. *Nat. Methods* **2016**, *13* (1), 55–58. https://doi.org/10.1038/nmeth.3658.

(10) Case, D. A.; Aktulga, H. M.; Belfon, K.; Ben-Shalom, I. Y.; Berryman, J. T.; Brozell, S. R.; Cerutti, D. S.; Cheatham III, T. E.; Cisneros, G. A.; Cruzeiro, V. W. D. *Amber 2023*; University of California, San Francisco, 2023.

(11) Michaud-Agrawal, N.; Denning, E. J.; Woolf, T. B.; Beckstein, O. MDAnalysis: A Toolkit for the Analysis of Molecular Dynamics Simulations. *J. Comput. Chem.* **2011**, *32* (10), 2319–2327. https://doi.org/10.1002/jcc.21787.

(12) Humphrey, W.; Dalke, A.; Schulten, K. VMD: Visual Molecular Dynamics. *J. Mol. Graph.* **1996**, *14* (1), 33–38. https://doi.org/10.1016/0263-7855(96)00018-5.

(13) Miller, B. R. I. I. I.; McGee, T. D. Jr.; Swails, J. M.; Homeyer, N.; Gohlke, H.; Roitberg, A. E. MMPBSA.Py: An Efficient Program for End-State Free Energy Calculations. *J. Chem. Theory Comput.* **2012**, *8* (9), 3314–3321. https://doi.org/10.1021/ct300418h.

(14) Mudasir; Wijaya, K.; Yoshioka, N.; Inoue, H. DNA Binding of Iron(II) Complexes with 1,10-Phenanthroline and 4,7-Diphenyl-1,10-Phenanthroline: Salt Effect, Ligand Substituent Effect, Base Pair Specificity and Binding Strength. *J. Inorg. Biochem.* **2003**, *94* (3), 263–271. https://doi.org/10.1016/S0162-0134(03)00007-2.

(15) Neese, F. Software Update: The ORCA Program System—Version 6.0. *WIREs Computational Molecular Science* **2025**, *15* (2), e70019. https://doi.org/10.1002/wcms.70019.

(16) Alberto, M. E.; Francés-Monerris, A. A Multiscale Free Energy Method Reveals an Unprecedented Photoactivation of a Bimetallic Os(II)-Pt(II) Dual Anticancer Agent. *Physical Chemistry Chemical Physics* **2022**, *24* (32), 19584–19594. https://doi.org/10.1039/d2cp02128e.

(17) Torrie, G. M.; Valleau, J. P. Nonphysical Sampling Distributions in Monte Carlo Free-Energy Estimation: Umbrella Sampling. *J. Comput. Phys.* **1977**, *23* (2), 187–199. https://doi.org/10.1016/0021-9991(77)90121-8.

(18) Grossfield, A. WHAM: The Weighted Histogram Analysis Method. http://membrane.urmc.rochester.edu/wordpress/?page_id=126 (accessed 2023-12-13).

(19) Dasari, S.; Bernard Tchounwou, P. Cisplatin in Cancer Therapy: Molecular Mechanisms of Action. *Eur. J. Pharmacol.* **2014**, *740*, 364–378. https://doi.org/10.1016/j.ejphar.2014.07.025.

(20) Seritan, S.; Bannwarth, C.; Fales, B. S.; Hohenstein, E. G.; Kokkila-Schumacher, S. I. L.; Luehr, N.; Snyder Jr., J. W.; Song, C.; Titov, A. V; Ufimtsev, I. S.; Martínez, T. J. TeraChem: Accelerating Electronic Structure and Ab Initio Molecular Dynamics with Graphical Processing Units. *J. Chem. Phys.* **2020**, *152* (22), 224110. https://doi.org/10.1063/5.0007615.

(21) Seritan, S.; Thompson, K.; Martínez, T. J. TeraChem Cloud: A High-Performance Computing Service for Scalable Distributed GPU-Accelerated Electronic Structure Calculations. *J. Chem. Inf. Model.* **2020**, *60* (4), 2126–2137. https://doi.org/10.1021/acs.jcim.9b01152.

(22) Seritan, S.; Bannwarth, C.; Fales, B. S.; Hohenstein, E. G.; Isborn, C. M.; Kokkila-Schumacher, S. I. L.; Li, X.; Liu, F.; Luehr, N.; Snyder Jr., J. W.; Song, C.; Titov, A. V; Ufimtsev, I. S.; Wang, L.-P.; Martínez, T. J. TeraChem: A Graphical Processing Unit-Accelerated Electronic Structure Package for Large-Scale Ab Initio Molecular Dynamics. *WIREs Computational Molecular Science* **2021**, *11* (2), e1494. https://doi.org/https://doi.org/10.1002/wcms.1494.

(23) Borrego-Sánchez, A.; Zemmouche, M.; Carmona-García, J.; Francés-Monerris, A.; Mulet, P.; Navizet, I.; Roca-Sanjuán, D. Multiconfigurational Quantum Chemistry Determinations of Absorption Cross Sections (σ) in the Gas Phase and Molar Extinction Coefficients (ε) in Aqueous Solution and Air–Water Interface. *J. Chem. Theory Comput.* **2021**, *17* (6), 3571–3582. https://doi.org/10.1021/acs.jctc.0c01083.
